# Suspect screening-based LC-HRMS characterization of short-chain peptide hydrolysates from marine macroalgae

**DOI:** 10.1007/s00216-026-06701-0

**Published:** 2026-08-01

**Authors:** Hind Achahbar, Andrea Cerrato, Enrique Durán-Guerrero, Ignacio Hernández, Aldo Laganà, Elena Lucà, Carmela Maria Montone, Enrico Taglioni, Anna Laura Capriotti

**Affiliations:** 1 Department of Analytical Chemistry, Faculty of Sciences, Institute for Viticultural and Agri-Food Research (IVAGRO), University of Cádiz, 11510 Puerto Real, Cádiz Spain; 2 Laboratory of Engineering Materials and Sustainable Energy (LEMSE), Faculty of Science, Abdelmalek Essaadi University, B.P. 2121, 93002 Tetouan, Morocco; 3 Department of Biology, University Marine Research Institute (INMAR), University of Cadiz, 11510 Puerto Real, Cadiz Spain; 4 Department of Chemistry, Sapienza University of Rome, Piazzale Aldo Moro 5, 00185 Rome, Italy; 5 Interuniversity Consortium INBB – Biostructures and Biosystems National Institute, Via Dei Carpegna 19, 00165 Rome, Italy

**Keywords:** Seaweed, Peptidomics, Marine biomass valorization

## Abstract

Marine macroalgae are increasingly recognized as sustainable and chemically diverse sources of valuable biomolecules. However, the short-chain peptide fraction (2–4 amino acids) of edible and invasive seaweeds remains poorly explored, mainly because conventional proteomics workflows are generally optimized for longer peptide sequences. In this study, an untargeted LC-HRMS suspect screening workflow was developed to characterize short-chain peptides generated by hydrolysis with Alcalase®, an alkaline protease from *Bacillus licheniformis *hydrolysis in 14 marine macroalgae species belonging to green, brown, and red phyla collected along the Atlantic coast of southern Spain. Overall, 333 short-chain peptides, predominantly dipeptides, were putatively annotated through accurate-mass filtering and manual MS/MS interpretation. Multivariate and univariate analyses revealed distinct peptide distribution patterns among algal groups, with brown algae showing a higher abundance of hydrophobic dipeptides associated with predicted bioactive properties according to in silico screening tools. Notably, invasive brown algae displayed peptide profiles largely comparable to edible species, supporting their potential valorization as sustainable sources of short-chain peptides. Exploratory in silico analyses further suggested the occurrence of peptide sequences potentially associated with antioxidant, antihypertensive, antidiabetic, sensory, and antifungal properties. Several peptides were also predicted to contribute to umami-related flavor attributes, highlighting the relevance of these matrices for future investigation of food-related peptide functionality. Although the proposed annotations and predicted bioactivities require orthogonal confirmation and experimental validation, the results provide a comprehensive exploratory overview of marine macroalgal short peptidomes. Overall, this study expands current knowledge on algae-derived short-chain peptides and demonstrates the applicability of untargeted LC-HRMS workflows for the characterization of underexplored peptide fractions in complex marine matrices.

**Supplementary Information:**

The online version contains supplementary material available at https://doi.org/10.1007/s00216-026-06701-0.

## Introduction

Marine macroalgae, commonly referred to as seaweeds, represent a highly diverse group of photosynthetic organisms classified into three major phyla based on their pigment composition: green algae (*Chlorophyta*), brown algae (*Phaeophyceae*), and red algae (*Rhodophyta*) [1]. These groups differ not only in their biochemical composition but also in their ecological distribution and metabolic adaptations, which ultimately influence the nature and abundance of their bioactive compounds [2]. Over recent years, seaweeds have attracted growing interest both as sustainable food sources and as reservoirs of bioactive compounds with potential health benefits [3] within the framework of the Blue Economy and circular bioeconomy strategies [4]. Their biomass contains a wide range of structurally diverse compounds, including polyphenols [5] and sulfated polyphenols [6], lipids [7, 8], polysaccharides [9], and pigments [10], all of which contribute to their nutritional value and highlight their potential in nutraceuticals and biotechnological innovation [11]. Marine macroalgae can be broadly classified into edible and invasive species. Edible seaweed species are widely valued for their distinctive chemical composition [7, 12]. In contrast, invasive seaweed species have received considerably less attention, despite their increasing ecological impact and rapid proliferation in coastal environments. Indeed, the uncontrolled proliferation of invasive seaweeds can promote algal blooms and excessive wrack accumulation along coastlines, disrupting aquatic ecosystems. The collection and valorization of both biomasses may help mitigate eutrophication by reducing nutrient loads, in line with the European Union Waste Framework Directive (Regulation 2008/98/EC) [13]. Beyond composting and biofuel production [14], invasive seaweeds are increasingly considered a valuable source of bioactive compounds.

Advances in nutraceutical research have promoted the development of personalized formulations based on precisely characterized bioactive compounds. These products are increasingly investigated for the management of specific disorders, including Metabolic Syndrome-related conditions, often as complementary approaches to conventional drug therapies [15]. Among the most promising compounds, bioactive short-chain peptides, particularly the ones derived from the enzymatic hydrolysis of plant-derived proteins, exhibit diverse physiological activities, making them promising candidates for various nutraceutical applications because of their typically higher bioavailability [16]. Although medium-sized peptides, including both tryptic and endogenous peptides, have been widely investigated over the last two decades in food science and clinical research [18–20], the study of short-chain peptides, generally consisting of 2 to 4 amino acids, has remained analytically challenging, and their systematic characterization in marine macroalgae remains extremely limited because standard proteomics approaches are inherently biased toward longer peptide sequences and often fail to capture short-chain peptides due to limitations in sample preparation, chromatographic retention, and database-driven identification strategies [18, 21,22].

Recent work from our research group has demonstrated that invasive marine macroalgae, such as *Gracilaria gracilis*, represent a rich source of both short- and medium-chain bioactive peptides obtained through enzymatic hydrolysis, with confirmed multifunctional properties including antioxidant, antihypertensive, and antidiabetic activities [23]. These findings highlighted the remarkable bioactive potential of algal-derived peptides and provided a strong analytical and biological foundation for further exploration of their low-molecular-weight fraction. High-resolution mass spectrometry (HRMS), particularly when coupled with advanced chromatographic separation and data-dependent acquisition (DDA) strategies, offers a powerful alternative for the detection and characterization of short-chain peptides. In this context, suspect screening approaches, combined with accurate mass measurements and MS/MS fragmentation analysis, can enable the annotation of short-chain peptide species that are otherwise inaccessible through traditional methodologies or de novo sequencing strategies [24]. Beyond analytical aspects, not only environmental conditions (e.g., salinity, nutrient concentrations, water movement, and sunlight exposure [25, 26] are considered key determinants of the molecular profile of marine macroalgae. It is largely documented that the production of bioactive compounds varies by algae species [27], but less attention has been given to peptides. In this context, a complete characterization of the short-chain peptide fraction in macroalgae belonging to different *phyla* could help elucidate patterns of species-specific peptidomic variation and clarify their possible functional significance.

Conventional bottom-up proteomics workflows are generally optimized for tryptic peptides characterized by higher molecular weights, multiple charge states, and extensive sequence-informative fragmentation. In contrast, short-chain peptides frequently exhibit limited chromatographic retention under reversed-phase conditions, simplified MS/MS fragmentation pathways, reduced sequence-specific fragment ion generation, and limited compatibility with conventional database-search algorithms. These analytical constraints considerably hinder their systematic characterization and highlight the need for dedicated HRMS-based workflows specifically tailored for low-molecular-weight peptidomics.

In the present paper, an untargeted LC-HRMS peptidomics workflow followed by suspect screening data processing was employed to assess the role of bioactive short-chain peptides generated via enzymatic hydrolysis with Alcalase®, a food-grade alkaline serine endopeptidase from Bacillus licheniformis, in discriminating 14 marine macroalgae collected from distinct coastal areas along the Atlantic shoreline of southern Spain (Cádiz region). Alcalase® was selected because of its broad substrate specificity and its well-documented efficiency in releasing low-molecular-weight bioactive peptides from different protein sources, including peptides with antioxidant, angiotensin-converting enzyme inhibitory, antidiabetic, anti-inflammatory, antimicrobial, and sensory-related properties. This makes it particularly suitable for the objectives of the present exploratory study, which aimed to generate and characterize short-chain peptide hydrolysates from underexplored algal biomass [28]

By combining HRMS with suspect screening approaches and multivariate and univariate data analysis, we sought to broaden the current understanding and characterization of short-chain peptides comprised in algal protein hydrolysates, investigate inter-*phylum* variations in peptide profiles, and evaluate algal biomass potential as a source of bioactive peptides for nutraceutical purposes.

## Materials and methods

### Chemicals

Trifluoroacetic acid (TFA) and Pierce bicinchoninic acid assay (BCA) Protein Assay Kit were bought from Thermo Fisher Scientific. Alcalase® enzyme from Bacillus licheniformis, TRIZMA® Hydrochloride, TRIZMA® base, sodium deoxycholate (SDC), Formic acid (FA) and all other chemicals and solvents were purchased from Sigma Aldrich (St. Louis, MO, USA). Optima® LC-MS grade water and acetonitrile (ACN) were purchased from Thermo Fisher Scientific (Waltham, MA, USA). Ultracel® 1 kDa Ultrafiltration Discs, regenerated cellulose (RC) 44.5 mm, and Durapore® (PVDF) 0.22 µm Membrane were bought from Merck Millipore (Tullagreen, Carrigtwohill, Co. Cork, IRL).

### Samples collection

**Fig. 1:**
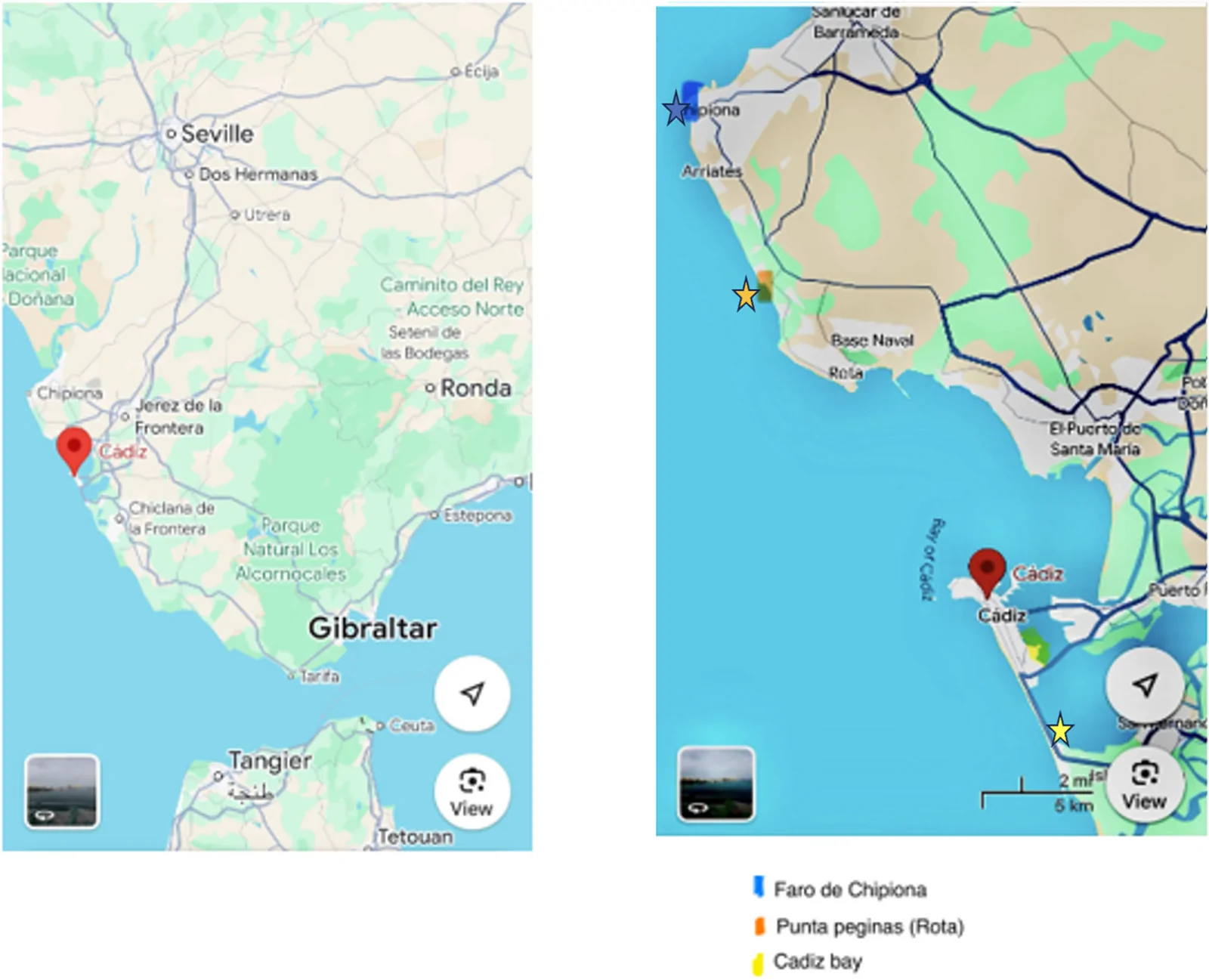
Geographic location of the seaweed sampling sites (Cadiz Bay Natural Park)along the Atlantic coast of the Gulf of Cádiz (southwestern Spain). The left panel shows the broader regional context, while the right panel provides a detailed view of the three sampling areas: lighthouse of Chipiona, Punta Peginas (Rota), and Santibáñez (Cadiz Bay Natural Park), where seaweed specimens were collected

Seaweed samples were collected in autumn (November and December 2025) along the Atlantic coast of the Gulf of Cadiz (southwestern Spain), encompassing three main coastal areas: outside wall of the lighthouse of Chipiona, rocky shores of Punta Peginas (Rota), and soft bottoms of Santibáñez (Cadiz Bay Natural Park). Data of the water quality form the sampling sites can be retrieved from https://dma.agenciamedioambienteyagua.es. The spatial distribution of the sampling sites is illustrated in Fig. 1.

Sampling was conducted during low tide by manual harvesting from the intertidal zone. The study focused on a selection of representative macroalgal species belonging to the three major taxonomic groups, green (*Chlorophyta*), brown (*Phaeophyceae)*, and red algae *(Rhodophyta*). Specifically, the investigated species included *Caulerpa prolifera*, *Codium fragile*, *Ulva rotundata*, *Chaetomorpha linum*, and *Blidingia minima* (Chlorophyta); *Sargassum vulgare*, *Rugulopteryx okamurae* (an invasive alien species), *Halopteris scoparia*, and *Gongolaria gibraltarica* (Phaeophyceae); and *Laurencia obtusa*, *Chondrophycus tenerrimus*, *Plocamium cartilagineum*, *Sphaerococcus coronopifolius*, and *Jania rubens* (Rhodophyta). The macroalgal species included in the quantitative dataset and used for the subsequent suspect screening and statistical analyses are listed in Table 1. Immediately after collection, samples were rinsed in situ with seawater to remove sand, epiphytes, and associated debris. Upon transport to the laboratory, samples were thoroughly washed with distilled water, freeze-dried, and stored at −20 °C until further processing. Taxonomic identification was performed based on morphological characteristics and supported, when necessary, by reference to updated taxonomic databases.

**Table 1:** Total protein content (%) determined in selected seaweed species using the BCA assay. Values represent the quantified protein levels obtained from each species under the experimental conditions described

Seaweed	Protein content (%)
*Caulerpa prolifera* (green)	3.3 ± 0.4%
*Codium fragile* (green)	1.10 ± 0.04%
*Ulva rotundata* (green)	1.6 ± 0.5%
*Chaetomorpha linum* (green)	9 ± 2%
*Blidingia minima* (green)	3.0 ± 0.5%
*Sargassum vulgare* (brown)	5.1 ± 0.3%
*Rugulopteryx okamurae* (brown)	2.0 ± 0.3%
*Halopteris scoparia* (brown)	6 ± 1%
*Gongolaria gibraltarica* (brown)	4 ± 2%
*Laurecia obtusa* (red)	2.3 ± 0.8%
*Chondrophycus tenerrimus* (red)	1.3 ± 0.2%
*Plocamium cartilagineum* (red)	2.4 ± 0.7%
*Sphaerococcus coronopifolius* (red)	4 ± 1%
*Jania rubens* (red)	4 ± 1%

Four independent biological replicates were analyzed for each macroalgal species. Each replicate underwent independent protein extraction, enzymatic hydrolysis, ultrafiltration, and LC-HRMS acquisition to ensure biological reproducibility and robustness of the comparative statistical analysis.

### Protein extraction and digestion

An aliquot of 100 mg from each of the 56 samples was weighed into 15 mL Falcon tubes. Each aliquot was extracted with 4 mL of buffer solution containing 50 mmol L^−1^ Tris-HCl at pH 8.5 and 2% (*m/v*) sodium deoxycholate (SDC), as previously optimized [29]. The samples were incubated on ice for 1 h, with vortexing for 1 min every 15 min, and then sonicated in an ultrasonic ice bath for another hour, these two steps were repeated twice in total. After that, the extracts were centrifuged at 8000 × g for 15 min at 4 °C, the supernatants were collected and stored in fridge at 4 °C until analysis. Protein content in the supernatants was determined using the Bicinchoninic Acid (BCA) Assay, with bovine serum albumin as the standard [30]. For each macroalgal species, the BCA assay was performed on the four independently extracted biological replicates, each measured in technical duplicate, and the protein content is reported as mean ± standard deviation (Table 1).

After quantification, the seaweed samples were subjected to hydrolysis with Alcalase® added at a 1:8 enzyme/protein ratio and incubated at 60 °C for 4 h. The digestion was stopped by acidifying the samples to pH 2 with trifluoroacetic acid (TFA). They were then centrifuged at 8000 × g for 15 min at room temperature to eliminate SDC. The total hydrolysate fraction was subjected to ultrafiltration using Ultracel® regenerated cellulose ultrafiltration discs with a 1 kDa nominal molecular weight limit (NMWL), 44.5 mm in diameter (Merck Millipore, Burlington, MA, USA). The filter was initially pre-treated and rehydrated in H₂O for 1 h and then completely dried using a Speed-Vac SC 250 Express (Thermo Savant, Holbrook, NY, USA). Before injection, the samples were reconstituted in 300 µL of H₂O/FA 99.9/0.1 (v/v) and filtered using Millex™ syringe-driven filter units with low protein-binding Durapore® PVDF 0.22 µm membranes (Merck Millipore, Burlington, MA, USA).

### Analysis of short-chain peptide mixture by ultra-high performance liquid chromatography-MS/MS

Short-chain peptide fractions obtained from ultrafiltration were analyzed by liquid chromatography coupled to high-resolution mass spectrometry (LC-HRMS) using a suspect screening approach, following previously established procedures [31]. Analyses were carried out on a Vanquish binary UHPLC system interfaced with a hybrid quadrupole-Orbitrap Q Exactive mass spectrometer (Thermo Fisher Scientific) equipped with a heated electrospray ionization (HESI) source. Chromatographic separation was achieved on a Kinetex XB-C18 column (100 × 2.1 mm, 2.6 μm particle size; Phenomenex) maintained at 40 °C. Data were acquired in positive ion mode over an m/z range of 150–750, with a resolving power of 70,000 (FWHM at m/z 200). An inclusion lists were generated using a custom script in Matlab (R2018), according to previously reported procedures [24]. Fragmentation data were collected in DDA mode, selecting the top five most intense precursor ions per scan cycle. Higher-energy collisional dissociation (HCD) was applied at a normalized collision energy of 35%, and MS/MS spectra were acquired at a resolution of 35,000 (FWHM at m/z 200). Each sample was analyzed in triplicate to ensure analytical reproducibility. Data processing was performed using Compound Discoverer 3.1 (Thermo Fisher Scientific) through a dedicated database developed in-house. specifically generated for di-, tri- and tetrapeptides. The database was compiled in MATLAB R2018a by combining the masses and molecular formulas of the 20 natural amino acids, according to our previously reported workflow [32]. The combination of natural amino acids resulted in 168,400 possible di-, tri- and tetrapeptide sequences. Since several sequences share the same elemental composition and exact mass, the list was filtered to remove duplicate masses, obtaining 4,980 unique m/z values. The complete sequence list was implemented in Compound Discoverer 3.1 (Thermo Fisher Scientific, Waltham, MA, USA), whereas the filtered list of unique masses was used to generate the inclusion lists for suspect screening LC-HRMS/MS acquisition.

The procedure included feature extraction from RAW files, retention time alignment, and removal of signals detected in blank samples or lacking associated MS/MS spectra. Further filtering steps were applied to retain only features matching the predefined mass list used during acquisition. Putative identification of short peptide sequences was achieved through manual interpretation of MS/MS spectra, supported by comparison with theoretical fragmentation patterns generated in silico using mMass [33]. The data presented are available at the following Zenodo Repository link: https://zenodo.org/records/21131643.

### Statistical analysis

Statistical analysis and data visualization were performed using MetaboAnalyst [34]. The data matrix was uploaded as a comma-separated values (CSV) file according to the platform guidelines. Data filtering was carried out using the interquartile range (IQR) method, while autoscaling was applied prior to statistical analysis. The processed datasets were subsequently used to generate volcano plots, box and whisker plot, and principal component analysis (PCA). One-way ANOVA and unpaired *t*-tests were performed using GraphPad Prism (GraphPad Prism 9, GraphPad Software, La Jolla, CA, USA). Statistical significance was assessed using a p-value threshold of 0.01. The resulting statistical outputs were used to identify significantly discriminant peptide sequences among the investigated macroalgal groups.

## Results and discussion

### Preliminary evaluation of macroalgal protein content

Before LC-HRMS peptide annotation, the total extractable protein content of the investigated macroalgae was evaluated using the BCA assay (Table 1), since protein availability represents a key parameter influencing the generation of peptide hydrolysates. Overall, the protein content showed a marked species-dependent variability, ranging from 1.10 ± 0.04% in *Codium fragile* to 9 ± 2% in *Chaetomorpha linum*. Among green algae, *Chaetomorpha linum* displayed the highest protein content, whereas *Codium fragile* showed the lowest value. Brown algae exhibited intermediate-to-high protein levels, with *Halopteris scoparia* and *Sargassum vulgare* showing relatively high protein contents, while *Rugulopteryx okamurae* presented a lower value. Red algae generally displayed intermediate protein contents, ranging from 1.3 ± 0.2% in *Chondrophycus tenerrimus* to approximately 4% in *Sphaerococcus coronopifolius* and Jania rubens. This variability is consistent with the well-known dependence of seaweed biochemical composition on species, taxonomic group, environmental conditions, and physiological status [35]

It should also be noted that some species showed higher variability among biological replicates, as reflected by larger standard deviations. This was particularly evident for *Gongolaria gibraltarica* (4 ± 2%), and to a lesser extent for species such as *Ulva rotundata* and *Laurencia obtusa*. Such lower repeatability is likely attributable to the intrinsic heterogeneity of wild-collected macroalgal biomass, including differences in thallus morphology, developmental stage, epiphyte removal efficiency, local microenvironmental exposure, and extraction efficiency. Therefore, the observed variability should not be interpreted only as analytical variability of the BCA assay, but also as a reflection of the biological heterogeneity of the collected algal material. For this reason, four independent biological replicates were processed for each species, each undergoing independent extraction, hydrolysis, ultrafiltration, and LC-HRMS analysis, in order to retain biological variability while ensuring robustness of the comparative peptidomic analysis.

### Short-chain peptides annotation

The proposed suspect screening pipeline enabled an extensive characterization of the short-chain peptide fraction across the investigated algal species. Short-chain peptides were annotated using a specialized processing workflow on Compound Discoverer 3.1, as previously described [32]. Overall, 332 short-chain peptides were putatively identified after manual interpretation of the MS/MS spectra, including 102 dipeptides, 174 tripeptides, and 56 tetrapeptides (Table S1). The Kinetex XB-C18 stationary phase was selected on the basis of previous short-chain peptidomics studies from our group [36, 37] in which this phase provided robust retention, reproducible chromatographic performance, and effective UHPLC-HRMS profiling of di-, tri-, and tetrapeptides. Nevertheless, due to the broad polarity range of short peptides, no single stationary phase can be considered fully comprehensive. Previous comparisons of C18 with porous graphitic carbon and HILIC separations demonstrated that these chromatographic mechanisms provide complementary selectivity and may increase peptide coverage. Therefore, the present C18-based method was intended as a robust comparative profiling platform for macroalgal hydrolysates, while orthogonal stationary phases could be explored in future studies to further expand the coverage of highly polar or poorly retained short peptides. As expected for very short peptide sequences, most annotated compounds eluted at early retention times, in agreement with their limited hydrophobic retention under reversed-phase conditions. However, the chromatographic method allowed the detection of both early-eluting polar peptides and more retained hydrophobic sequences, supporting its suitability for comparative short-chain peptide profiling. A representative full-scan base peak chromatogram is reported in Figure S1.

According to an adapted confidence-level classification based on Schymanski/MSI criteria, these annotations should be considered confidence level 2 identifications since they were supported by accurate mass measurements and diagnostic MS/MS fragmentation patterns but were not confirmed using authentic analytical standards [38]. Table S1 reports the identification data for the tentatively annotated sequences. Since leucine (Leu) and isoleucine (Ile) are isobaric residues that cannot be unequivocally discriminated using conventional MS^2^ fragmentation data alone, peptide annotations containing these amino acids were classified as confidence level 3 identifications according to adapted Schymanski/MSI criteria. Therefore, the three-letter notation “Xle” and the one-letter code “J” were employed to indicate either Leu or Ile residues. In particular, the putatively identified sequences spanned di-, tri-, and tetrapeptides, with a predominance of dipeptides, consistent with the applied ultrafiltration cutoff and enzymatic hydrolysis conditions. Indeed, enzymatic hydrolysis with Alcalase® is considered a straightforward process that can be easily controlled and optimized to produce a hydrolysate rich in short-chain bioactive peptides, as smaller peptides are more likely to reach the bloodstream and target organs intact [17]. The putatively identified short-chain peptides covered a broad compositional and physicochemical space, as reflected by differences in amino acid composition, sequence arrangement, peptide length, and GRAVY index values reported in Table S1. In this context, structural diversity refers to the variability in residue combinations and sequence order among di-, tri-, and tetrapeptides, rather than to large conformational or higher-order structural features. This molecular diversity highlights the remarkable biochemical complexity of algal-derived hydrolysates and supports their potential as reservoirs of structurally diverse peptide sequences associated with predicted bioactivities. From a compositional perspective, the dataset revealed the recurrent presence of small hydrophobic and neutral amino acids, especially Val, Ala, Gly, Xle (Leu/Ile), Pro, Phe, Tyr, and Met, indicating that the peptide pool is largely hydrophobic to amphipathic in character. The predominance of hydrophobic and amphipathic peptide motifs is particularly relevant from a nutraceutical perspective, since these structural features are frequently associated with antioxidant, antihypertensive, and multifunctional bioactive properties [39, 40].

### Comparative statistical analysis of aacroalgal peptidomes

**Fig. 2:**
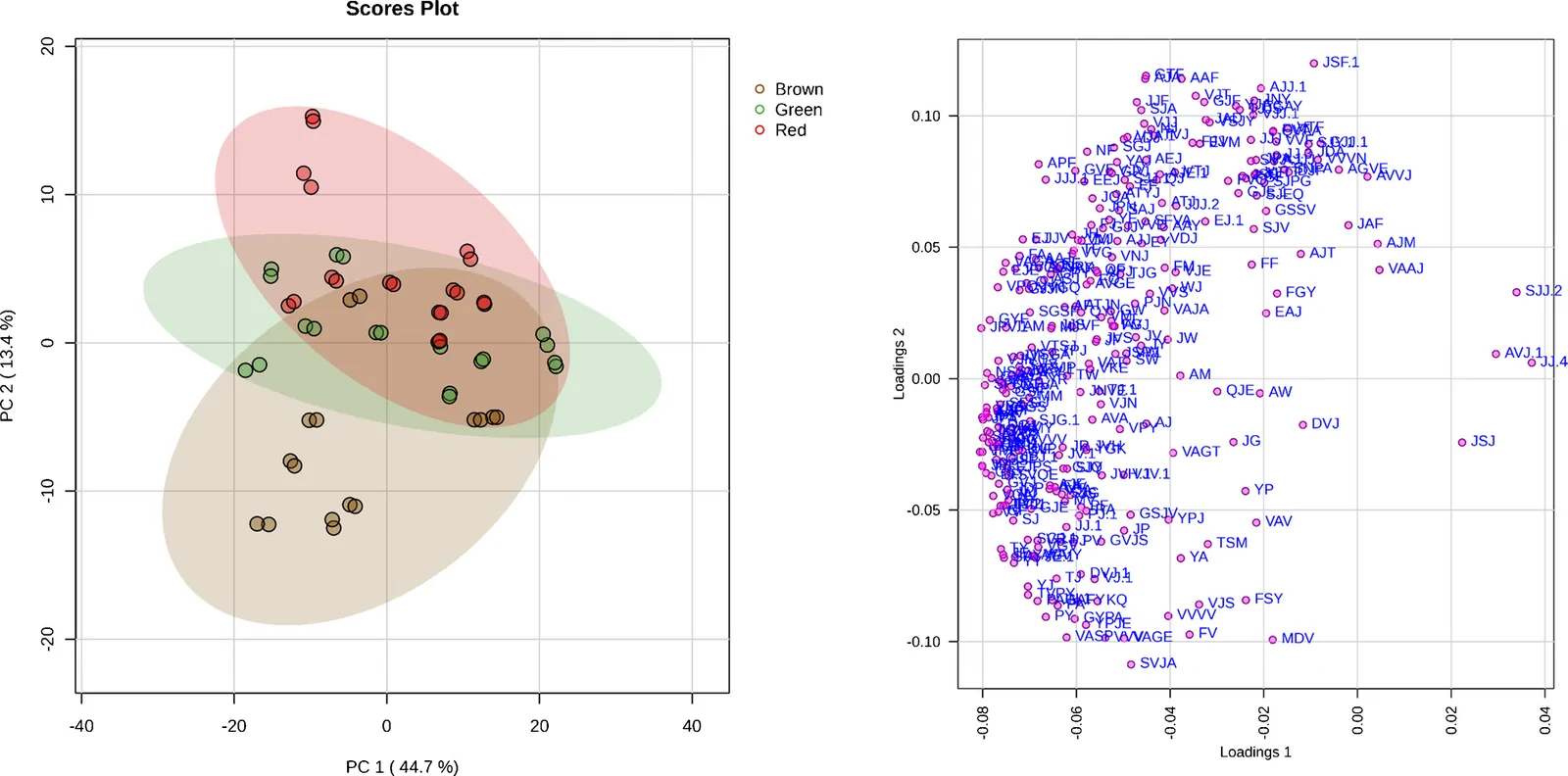
Principal component analysis (PCA) score plot (left) and corresponding loadings plot (right) obtained from the normalized peak areas of all identified peptide sequences in brown, green, and red macroalgae samples

Principal Component Analysis (PCA) performed on the normalized peak areas of all 332 annotated peptide sequences revealed a certain degree of clustering trend among the different macroalgal groups (brown, green, and red algae) (Fig. 2).

It should be noted that PCA was used here as an exploratory and unsupervised tool to visualize the main sources of variance in the peptide dataset, rather than to develop a predictive classification model. Therefore, the observed clustering trends were interpreted together with univariate statistical outputs, avoiding supervised modeling approaches that could overfit the data given the exploratory nature of the study and the limited sample size relative to the number of peptide variables.

**Fig. 3:**
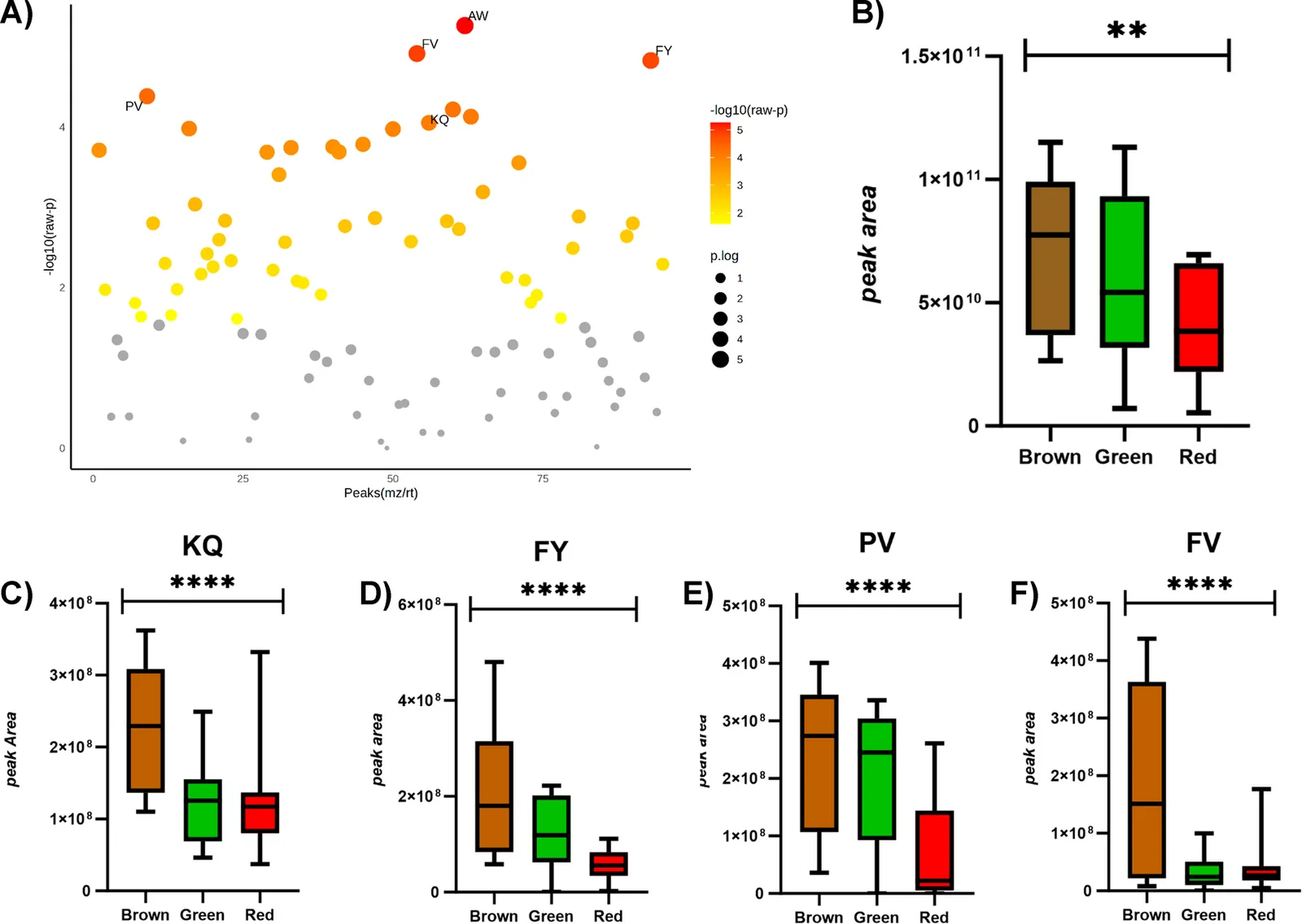
Statistical analysis of dipeptide abundance in brown, green, and red macroalgae. **A** Volcano-style plot showing the significance distribution of identified dipeptides obtained from one-way ANOVA performed on the dipeptide-only data matrix; selected significantly discriminant dipeptides are highlighted. **B** Box-and-whisker plot representing the overall distribution of normalized dipeptide peak areas among algal groups, showing a higher dipeptide content in brown algae. **C**–**F** Representative box-and-whisker plots of significantly different dipeptide sequences (KQ, FY, PV, and FV) identified by ANOVA. Statistical significance is indicated by the following: *p < 0.1; **p < 0.01; ***p < 0.001; ****p < 0.0001

The first two principal components explained a substantial proportion of the total variance (PC1 44.7%, PC2 13.4%), highlighting differences in peptide composition among species. Brown algae showed a more distinct distribution pattern compared to red and green algae. Inspection of the corresponding loadings plot indicated that many peptides, especially dipeptides, were strongly associated with the brown algal samples. Since short dipeptides are widely recognized for their potential bioactive properties, these observations suggested that brown algae may represent a particularly rich source of structurally diverse short-chain peptides. To further investigate the distribution of short-chain peptides, we focused specifically on the dipeptide fraction by extracting a second data matrix containing only identified dipeptides. Multivariate PCA resulted in a higher proportion of the total variance explained by the first two components (PC1 55.9%, PC2 11.8%) and a clearer clustering of brown algae (Figure S2). Later, one-way ANOVA was performed to evaluate differences among brown, green, and red macroalgae. As shown in the significance plot (Fig. 3A), several dipeptides exhibited statistically significant differences in abundance across the algal groups, with many of the most significant features associated with brown algae. The overall distribution of normalized dipeptide peak areas (Fig. 3B) indicated that brown algae generally contained higher dipeptide levels compared to green and red species.

To illustrate these trends, selected examples of significantly different dipeptides are reported in the box-and-whisker plots (Fig. 3C–-F), including KQ, FY, TY, and FV.

**Fig. 4:**
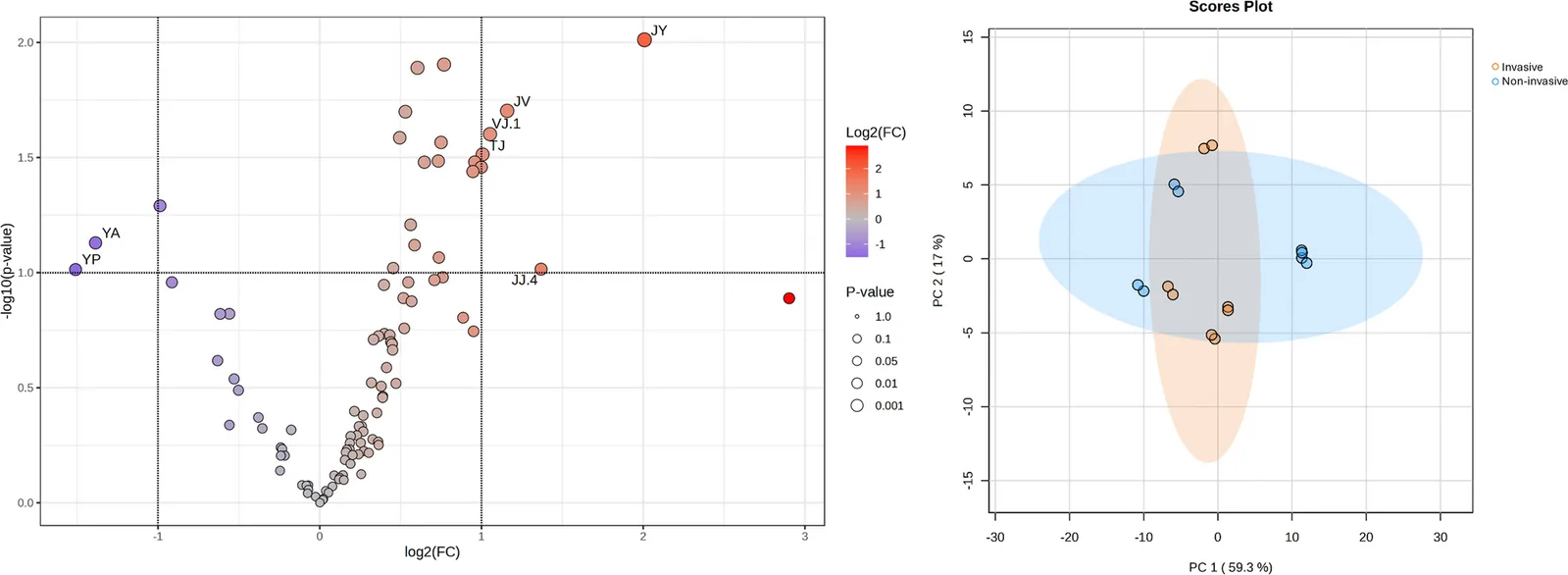
Volcano plot highlighting differential dipeptide abundance between invasive and edible brown algae (left). Positive log2(FC) values indicate dipeptides more abundant in invasive species, while negative values indicate higher abundance in edible species. Multivariate and differential analysis of dipeptide profiles in edible and invasive brown algae. PCA score plot based exclusively on the dipeptide data matrix (right), showing the distribution of edible and invasive brown algae samples according to their dipeptide composition

These representative sequences showed significantly higher abundances in brown algae, supporting the hypothesis that brown macroalgae are enriched in potentially bioactive dipeptides relative to the other algal groups. Based on these findings, the subsequent analyses were focused exclusively on brown algae, still using a data matrix composed only of 102 dipeptides, to compare native and invasive species. The PCA score plot shows a substantial overlap between edible and invasive brown algae samples, indicating the absence of a strong statistical separation between the two groups based on their dipeptide composition (Fig. 4).

The partial overlap between the two groups indicates the absence of a strong statistical separation, suggesting a highly comparable dipeptide profile between edible and invasive species. PC1 and PC2 explain 59.3% and 17.0% of the total variance, respectively.

This result supports the hypothesis that invasive brown algae may represent a valuable and sustainable source of bioactive dipeptides. Therefore, based on our results, biomass derived from algal blooms could be effectively valorized through recovery strategies aimed at producing formulations enriched in bioactive compounds, contributing both to environmental management and to the development of candidate peptide-enriched hydrolysates for future functional investigations. The volcano plot (Fig. 4) further supports this interpretation. Since the analysis was performed exclusively on dipeptides, the observed differences specifically reflect variations in dipeptide abundance between invasive and edible brown algae. Compounds such as JY, JV, VJ, and TJ appear markedly upregulated in invasive samples, whereas only a limited number of dipeptides are more abundant in edible algae. Overall, the volcano plot reveals a general trend toward higher abundance of specific dipeptides in invasive brown algae, reinforcing their potential as an alternative and sustainable source of bioactive compounds for nutraceutical applications.

### Distribution of essential amino acid-containing short-chain peptide sequences in red, green, and brown algae

From a nutraceutical point of view, the amino acid (AA) composition of protein hydrolysates is commonly evaluated as it is considered one of the most important aspects influencing the formulations’s nutritional value, as peptides rich in essential amino acids (EAAs) contribute to meeting the daily dietary requirements of humans [41]. The focus on EAA-containing short-chain peptides is particularly relevant in the nutraceutical field, as bioactive peptides enriched in essential amino acids have been associated with several health-promoting properties, including antioxidant, antihypertensive, antimicrobial, anti-inflammatory, and metabolic regulatory activities [42, 43]. In fact, short-chain peptides intrinsic enhanced gastrointestinal stability, absorption efficiency, and bioavailability do not only rely on sequence length, but they strongly depend on AA composition and sequence. Consequently, identifying specific EAA-containing peptide signatures may provide valuable insight into the nutraceutical potential and functional properties of algae-derived protein hydrolysates. We subsequently filtered the data matrix to retain only short-chain peptide sequences containing EAAs together with their relative abundances across all samples. Specifically, sequences containing methionine (M), phenylalanine (F), tyrosine (Y), tryptophan (W), threonine (T), and lysine (K) were selected for further analysis. In contrast, isoleucine (I) and leucine (L) were excluded for two main reasons: first, these isomeric amino acids cannot be reliably distinguished using conventional MS/MS approaches; and second, they were highly abundant across most detected sequences, potentially limiting their specificity and biological relevance. The multivariate and univariate analyses of EAA-containing short-chain peptides revealed distinct patterns depending on the amino acid class considered. PCA performed on the data matrix comprising M- and K-containing peptides showed extensive overlap among the brown, green, and red algal groups, with no statistically significant discrimination observed (Figure S3A–D). Although minor clustering tendencies were visible along PC1 and PC2, the broad overlap of confidence ellipses and the non-significant ANOVA results indicate that M- and K-containing peptide profiles were relatively conserved across the investigated algae species. This suggests that these peptide classes may represent common structural or metabolic features shared among the taxa rather than species-specific bioactive signatures. In contrast, the analysis of F-, Y-, W-. and T-containing peptides revealed a markedly different pattern and ANOVA identified significant differences among algal groups, indicating that aromatic EAA-containing peptides contributed strongly to species discrimination. The PCA and subsequent statistical analyses demonstrated that several F-, Y-, W- and T-containing peptide sequences were differentially abundant across brown, green, and red algae, supporting the hypothesis that aromatic amino acid-enriched peptides are more taxonomically and functionally informative (Figure S4A–D). This observation is particularly relevant from a nutraceutical perspective because peptides containing aromatic residues such as F, Y, and W are frequently associated with antioxidant and radical-scavenging activities due to their electron-donating and resonance-stabilizing properties [44], while T-containing peptides may contribute to immune modulation and metabolic regulation [45]. The univariate analyses further highlighted specific peptide sequences exhibiting strong statistical significance, as reflected by elevated − log10(*p*-value) scores and significant ANOVA outcomes. In the F-containing peptide dataset, sequences such as FT, DJF, JF, and VVF were among the representative peptides showing significant differential abundance, with several aromatic peptide sequences exhibiting higher relative abundances in green algae, suggesting a potential enrichment of antioxidant-related peptide motifs in this group (Figure S5A–F). Green algae species were also characterized by statistically significant production of W-containing short-chain sequences, with WN, AW, JW, and VW exhibiting significant differential abundance (Figure S6A–F). Similarly, within the Y-containing peptide dataset, sequences including FSY, AY, YA, and YPJE showed highly significant intergroup variation, with several peptides markedly enriched in brown algae (Figure S7A–F). Finally, T-containing short-chain peptide sequences appeared to be particularly enriched in red algal species, as evidenced by their higher relative abundances within this phylum (Figure S8A–F). Overall, these findings suggest that while M- and K-containing peptides do not provide strong discriminatory power among algal species, aromatic amino acid-containing peptides, particularly those enriched in F and Y residues, represent more distinctive biochemical signatures within the generated protein hydrolysates. Their differential abundance across algae groups likely reflects differences in the original protein composition and susceptibility to enzymatic hydrolysis among the algal species. From a nutraceutical perspective, these results are particularly relevant because they may support the development of plant-based peptide-enriched formulations with targeted functional properties. Identifying algal species that preferentially generate hydrolysates enriched in specific essential amino acid-containing peptides could therefore facilitate the selection of optimal raw materials to produce tailored nutraceutical ingredients with enhanced antioxidant, metabolic, or health-promoting potential.

### In silico assessment of predicted bioactivities, sensory properties, antifungal potential, and antioxidant capacity of short-chain peptides from the invasive alien brown species

The following analyses should be interpreted as exploratory prioritization strategies aimed at identifying candidate peptide sequences potentially associated with bioactive and sensory properties.

Table S2 presents the in silico bioactivity and sensory characterization of the 30 short-chain peptide sequences showing the highest mean normalized peak areas within invasive brown algal samples. This subset was not selected as a statistical marker panel, but rather as an exploratory prioritization of the most abundant peptides in the invasive brown biomass for subsequent bioactivity and sensory prediction. These peptides were selected following multivariate analysis, where brown algae exhibited a more distinct peptide distribution pattern compared with red and green algae (Fig. 2). Since no statistically significant differences were observed between invasive and non-invasive brown algae, the invasive biomass was prioritized because of its potential for environmental reuse and valorization. The selected peptides were screened using several open-access peptide databases and prediction tools, including BIOPEP-UWM [46], AHTPDB [47], Bioactive Peptides, TastePeptidesDB, Umami_YYDS, Umami_IP [48], AntiFungiPept Predictor [49], and AnOxPePred [50].

The results suggest that the peptide fraction from invasive brown algae is enriched in multifunctional short peptides with predicted antihypertensive, antidiabetic, antioxidant, sensory, and antifungal activities. Several peptides, including VY, YJ, AY, JY, VV, and SF, were particularly notable because they combined multiple predicted activities, since they were identified as known bioactive sequences in BIOPEP-UWM and AHTPDB, particularly as angiotensin-converting enzyme (ACE) inhibitors and dipeptidyl peptidase-IV (DPP-IV) inhibitors. These activities are commonly associated with antihypertensive and antidiabetic effects, respectively, suggesting that invasive brown algae may represent a promising source of health-promoting peptides. These peptides also exhibited some of the highest free radical scavenging (FRS) scores, with JY (0.502), YJ (0.494), AY (0.492), and VY (0.489) showing the strongest predicted antioxidant potential, aligned with the enhanced radical scavenging capacity of aromatic amino acids-containing peptides. The sensory analysis revealed that bitterness was the dominant predicted taste attribute among the identified peptides. Many hydrophobic short-chain peptides, particularly those enriched in V, Y, A, or F residues, were classified as bitter by both BIOPEP and TastePeptidesDB. This observation is consistent with previously reported relationships between peptide hydrophobicity and bitter taste perception [51]. In contrast, several peptides, including JGVS, GVJ, GJE, GTVA, ATJ, and SF, were predicted to possess umami characteristics, with VV and GVJ showing the highest umami intensity scores. These findings suggest that invasive brown algae may also contain flavor-enhancing peptides with potential applications in functional foods or food formulation. The simultaneous occurrence of predicted bioactive and flavor-related peptides further supports the potential exploitation of invasive brown algae as multifunctional ingredients for future food.

Although these analyses remain predictive and require experimental validation, they provide preliminary insights into the multifunctional potential of algae-derived short peptides.

The antifungal prediction analysis further highlighted the biofunctional potential of these peptides. Several sequences displayed high probabilities of antifungal activity, particularly JY, AJJJ, VJ, GVJ, VY, and YJ, with prediction probabilities exceeding 99% in some cases. In addition, some peptides demonstrated elevated predicted activity against specific fungal species, especially *Candida neoformans* and *Candida albicans*. The differences between antifungal prediction probabilities and AFI values arise because they capture distinct aspects of peptide behavior. The antifungal probability typically reflects how similar a sequence is to known antifungal peptides based on classification models, while AFI is a physicochemical index estimating potential functional potency based on properties such as charge, hydrophobicity, and amphipathicity. As a result, some peptides show high antifungal probability but low AFI because they resemble known antifungal motifs but lack strong membrane-active characteristics, whereas others display high AFI but low probability because they possess strong physicochemical features associated with antifungal activity but are not similar to sequences in the training dataset. Therefore, reliable identification of potential antifungal peptides should rely on a combined approach that considers both measures, prioritizing sequences where both metrics agree while also allowing high-AFI outliers for possible novel candidates, alongside basic physicochemical constraints. Although these predictions require experimental validation, they suggest that peptides derived from invasive brown algae may possess antimicrobial properties of potential interest for nutraceutical, pharmaceutical, or food preservation applications. Considering that *Rugulopteryx okamurae* is currently causing severe ecological and socioeconomic impacts along the southern Spanish coastline, including biodiversity loss, alteration of native benthic communities, and major accumulation events affecting coastal ecosystems and human activities [52], any additional knowledge regarding its molecular composition and functional properties contributes added value toward the sustainable valorization of this invasive biomass. Overall, these findings support the potential valorization of invasive species as sustainable and underexplored sources of multifunctional short-chain bioactive peptides. The enrichment of small peptides, especially dipeptides, together with their predicted antioxidant, antihypertensive, antidiabetic, sensory, and antifungal properties, supports the potential valorization of nuisance algal biomass within sustainable biorefinery and circular bioeconomy approaches.

The present study remains exploratory in nature and relies on putative peptide annotation supported by manual MS/MS interpretation and predictive in silico tools. Therefore, orthogonal structural confirmation and experimental validation of the proposed bioactivities will be required in future studies to confirm the functional relevance of the identified sequences.

## Conclusions

The proposed LC-HRMS suspect screening workflow enabled an extensive characterization of the short-chain peptide fraction generated from edible and invasive marine macroalgae, highlighting the remarkable molecular diversity of algal-derived hydrolysates. The comparative peptidomic analysis revealed distinct peptide distribution patterns among green, red, and brown algae, with brown species showing a higher abundance of short hydrophobic dipeptides potentially associated with bioactive properties. Notably, invasive brown algae exhibited peptide profiles largely comparable to edible species, supporting their potential valorization as sustainable and underexplored sources of nutritionally relevant short peptides. The in silico analyses further suggested that several identified peptides may possess multifunctional properties, including predicted antioxidant, antihypertensive, antidiabetic, sensory, and antifungal activities, while also revealing the presence of sequences potentially contributing to umami-related flavor attributes. Although these findings remain predictive and require experimental validation, they provide valuable preliminary insights into the possible functional and nutraceutical relevance of algae-derived short peptides. Overall, this study contributes to expanding the current knowledge of short-chain peptidomes in marine macroalgae and supports the sustainable exploitation of nuisance algal biomass within circular bioeconomy and future food strategies. The identification of peptide-enriched hydrolysates from invasive seaweeds may open new perspectives for the development of functional ingredients and peptide-based nutraceutical formulations derived from marine resources. The present study remains exploratory in nature and relies on putative peptide annotation supported by manual MS/MS interpretation and predictive in silico tools. Therefore, orthogonal structural confirmation and experimental validation of the proposed bioactivities will be required in future studies to confirm the functional relevance of the identified sequences.

## Supplementary Information

Below is the link to the electronic supplementary material.

Supplementary file1 (DOCX 2.61 MB)

Supplementary file2 (CSV 7.10 MB)

## Data Availability

The data presented are available at the following Zenodo Repository link: https://zenodo.org/records/21131643.
