# Supplementary material for: Suspect screening-based LC-HRMS characterization of short-chain peptide hydrolysates from marine macroalgae
Source: Anal Bioanal Chem. 2026 Aug 1;418(21):7051–63. doi: 10.1007/s00216-026-06701-0 (PMC13658488; doi:10.1007/s00216-026-06701-0)
Supplement: Supplementary file 1 [file 216_2026_6701_MOESM1_ESM.docx]

**Supplementary Material**

**Suspect screening-based LC-HRMS characterization of short-chain peptide hydrolysates from marine macroalgae**

Hind Achahbar^a,b^, Andrea Cerrato^d,e^, Enrique Durán-Guerrero^a^, Ignacio Hernández Carrero^c^, Aldo Laganà^d,e^ Elena Lucà^d^, Carmela Maria Montone^d,e^, Enrico Taglioni^d,e^, Anna Laura Capriotti^d,e^*

^a^Department of Analytical Chemistry, Faculty of Sciences, Institute for Viticultural and Agri-food Research (IVAGRO), University of Cádiz, E-11510 Puerto Real, Cádiz, Spain

^b^Laboratory of Engineering Materials and Sustainable Energy (LEMSE), Faculty of Science, Abdelmalek Essaadi University, B.P. 2121, 93002, Tetouan, Morocco

^c^Department of Biology, University Marine Research Institute (INMAR), University of Cadiz, E-11510 Puerto Real, Cadiz, Spain

^d^Department of Chemistry, Sapienza University of Rome, Piazzale Aldo Moro 5, 00185 Rome, Italy

^e^Interuniversity Consortium INBB – Biostructures and Biosystems National Institute, Via dei Carpegna 19, 00165, Rome, Italy

***Corresponding author**:

Prof. Anna Laura Capriotti

Department of Chemistry, Sapienza Università di Roma, Piazzale Aldo Moro 5, 00185 Rome, Italy

E-mail: annalaura.capriotti@uniroma1.it

Telephone: (+39) 06 4991 3062

Fax (+39) 06 4906 31

**Figure S1**. Representative full-scan base peak chromatogram of a matrix-matched quality control (QC) sample run, illustrating the overall chromatographic profile of short-chain peptides under the applied analytical conditions.

**Figure S2.** Principal component analysis (PCA) score plot obtained from the normalized peak areas of all identified dipeptides in brown, green, and red macroalgae samples.

**Figure S3**. Principal component analysis (PCA) score plot Box-and-whisker plot of the relative abundance of M-containing (A-B) and K-containing (C-D) essential amino acids in the three sample groups (brown, green, and red). No statistically significant differences were observed among groups (“ns”, p > 0.05). The PCA score plot (PC1 = 50%, PC2 = 13.1%) shows substantial overlap among the three groups, indicating the absence of clear clustering or discrimination based on the analyzed amino acid profile.

**Figure S4**. Principal component analysis (PCA) score plots of peptide profiles grouped according to the presence of specific amino acid residues: F-containing peptides (A), Y-containing peptides (B), T-containing peptides (C), and W-containing peptides (D). Samples are classified into brown, green, and red groups.

**Figure S5**. Differential analysis of F-containing peptides among brown, green, and red sample groups. Volcano plot showing peptide features ranked according to statistical significance (A), where selected discriminant peptides are annotated. Point color and size reflect increasing statistical significance. Box-and-whisker plot showing the relative abundance (%) of F-containing peptides across the three groups, revealing significant differences among groups (**, p < 0.01) (B). Panels (C-F) show the peak area distributions of selected discriminant peptides: FT (C), DJF (D), JF (E), and VVF (F). Green samples generally exhibited higher peptide abundance compared with brown and red groups. Statistical significance is indicated as follows: ***, p < 0.001 and ****, p < 0.0001.

**Figure S6**. Differential analysis of W-containing peptides among brown, green, and red sample groups. Volcano plot showing peptide features ranked according to statistical significance (A), where selected discriminant peptides are annotated. Point color and size reflect increasing statistical significance. Box-and-whisker plot showing the relative abundance (%) of W-containing peptides across the three groups, revealing significant differences among groups (***, p < 0.001) (B). Panels (C-F) show the peak area distributions of selected discriminant peptides: WN (C), AW (D), JW (E), and VW (F). Green samples generally exhibited higher peptide abundance compared with brown and red groups. Statistical significance is indicated as follows: **, p < 0.01 and ****, p < 0.0001.

**Figure S7**. Differential analysis of Y-containing peptides among brown, green, and red sample groups. Volcano plot showing peptide features ranked according to statistical significance (A), where selected discriminant peptides are annotated. Point color and size reflect increasing statistical significance. Box-and-whisker plot showing the relative abundance (%) of Y-containing peptides across the three groups, revealing significant differences among groups (***, p < 0.001) (B). Panels (C-F) show the peak area distributions of selected discriminant peptides: FSY (C), AY (D), YA (E), and YPJE (F). Brown samples generally exhibited higher peptide abundance compared with green and red groups. Statistical significance is indicated as follows: ***, p < 0.001 and ****, p < 0.0001.

**Figure S8**. Differential analysis of T-containing peptides among brown, green, and red sample groups. Volcano plot showing peptide features ranked according to statistical significance (A), where selected discriminant peptides are annotated. Point color and size reflect increasing statistical significance. Box-and-whisker plot showing the relative abundance (%) of T-containing peptides across the three groups, revealing significant differences among groups (**, p < 0.01) (B). Panels (C-F) show the peak area distributions of selected discriminant peptides: GTF (C), VJTT (D), VJT (E), and TJJS (F). Red samples generally exhibited higher peptide abundance compared with brown and green groups. Statistical significance is indicated as follows: ***, p < 0.001 and ****, p < 0.0001.

**Table S1**. Identification data of the short-chain peptide sequences annotated in the algal protein hydrolysates, comprising compound identity based on the three and one-letter nomenclature, RT, molecular formula, experimental and calculated M+H, mass error (ppm),and *GRAVY index score.

| ID (1 letter, J = I/L) | Formula | Theoretical [M+H]^+^ | Experimental [M+H]^+^ | Δmass (ppm) | RT [min] | Length | GRAVY |
| --- | --- | --- | --- | --- | --- | --- | --- |
| KQ | C11H22N4O4 | 275.1719 | 275.1716 | 1.18 | 0.73 | 2 | -3.7 |
| RA | C9H19N5O3 | 246.1566 | 246.1564 | 1.02 | 0.74 | 2 | -1.35 |
| VR | C11H23N5O3 | 274.1879 | 274.1878 | 0.51 | 0.82 | 2 | -0.15 |
| JN | C10H19N3O4 | 246.1454 | 246.1453 | 0.15 | 0.93 | 2 | 0.325 |
| APA | C11H19N3O4 | 258.1454 | 258.1453 | 0.3 | 0.93 | 3 | 0.667 |
| JR | C12H25N5O3 | 288.2036 | 288.2035 | 0.28 | 0.93 | 2 | -0.175 |
| YGK | C17H26N4O5 | 367.1981 | 367.1981 | 0.11 | 0.95 | 3 | -1.867 |
| KY | C15H23N3O4 | 310.1767 | 310.1766 | 0.25 | 0.97 | 2 | -2.6 |
| JS | C9H18N2O4 | 219.1345 | 219.1345 | 0.04 | 0.98 | 2 | 1.675 |
| KVP | C16H30N4O4 | 343.2345 | 343.2344 | 0.28 | 1.00 | 3 | -0.433 |
| JH | C12H20N4O3 | 269.1614 | 269.1613 | 0.3 | 1.03 | 2 | 0.475 |
| YAR | C18H28N6O5 | 409.2199 | 409.2199 | 0.05 | 1.03 | 3 | -1.333 |
| EPT | C14H23N3O7 | 346.1614 | 346.1613 | 0.24 | 1.04 | 3 | -1.933 |
| FK | C15H23N3O3 | 294.1818 | 294.1817 | 0.31 | 1.06 | 2 | -0.55 |
| AM | C8H16N2O3S | 221.096 | 221.0959 | 0.2 | 1.09 | 2 | 1.85 |
| YR | C15H23N5O4 | 338.1828 | 338.1827 | 0.25 | 1.10 | 2 | -2.9 |
| VAT | C12H23N3O5 | 290.1716 | 290.1716 | -0.06 | 1.15 | 3 | 1.767 |
| PV | C10H18N2O3 | 215.1396 | 215.1395 | 0.11 | 1.16 | 2 | 1.3 |
| AVA | C11H21N3O4 | 260.161 | 260.161 | 0.03 | 1.18 | 3 | 2.6 |
| YE | C14H18N2O6 | 311.1243 | 311.1243 | 0.12 | 1.19 | 2 | -2.4 |
| AJN | C13H24N4O5 | 317.1825 | 317.1825 | 0.13 | 1.20 | 3 | 0.817 |
| VVS | C13H25N3O5 | 304.1872 | 304.1872 | 0.04 | 1.22 | 3 | 2.533 |
| JE | C11H20N2O5 | 261.145 | 261.145 | 0.36 | 1.24 | 2 | 0.325 |
| YA | C12H16N2O4 | 253.1188 | 253.1187 | 0.47 | 1.24 | 2 | 0.25 |
| VVQ | C15H28N4O5 | 345.2138 | 345.2138 | 0.12 | 1.25 | 3 | 1.633 |
| VAGT | C14H26N4O6 | 347.1931 | 347.1929 | 0.42 | 1.25 | 4 | 1.225 |
| TGV | C11H21N3O5 | 276.1559 | 276.156 | -0.07 | 1.26 | 3 | 1.033 |
| PAGA | C13H22N4O5 | 315.1668 | 315.1667 | 0.42 | 1.26 | 4 | 0.4 |
| JA | C9H18N2O3 | 203.1396 | 203.1396 | 0.01 | 1.27 | 2 | 2.975 |
| AVGE | C15H26N4O7 | 375.188 | 375.1878 | 0.48 | 1.30 | 4 | 0.525 |
| VGA | C10H19N3O4 | 246.1454 | 246.1454 | 0.07 | 1.33 | 3 | 1.867 |
| DVA | C12H21N3O6 | 304.1509 | 304.1508 | 0.15 | 1.33 | 3 | 0.833 |
| YSGS | C17H24N4O8 | 413.1672 | 413.1671 | 0.28 | 1.35 | 4 | -0.825 |
| VKE | C16H30N4O6 | 375.2244 | 375.2242 | 0.52 | 1.35 | 3 | -1.067 |
| JG | C8H16N2O3 | 189.1239 | 189.1236 | 1.44 | 1.36 | 2 | 1.875 |
| JE | C11H20N2O5 | 261.145 | 261.1449 | 0.43 | 1.37 | 2 | 0.325 |
| VVG | C12H23N3O4 | 274.1767 | 274.1766 | 0.32 | 1.39 | 3 | 2.667 |
| JVH | C17H29N5O4 | 368.2298 | 368.2296 | 0.39 | 1.41 | 3 | 1.717 |
| VAGA | C13H24N4O5 | 317.1825 | 317.1825 | 0.03 | 1.43 | 4 | 1.85 |
| SY | C12H16N2O5 | 269.1137 | 269.1136 | 0.46 | 1.45 | 2 | -1.05 |
| VVGG | C14H26N4O5 | 331.1981 | 331.1979 | 0.73 | 1.45 | 4 | 1.9 |
| VASP | C16H28N4O6 | 373.2087 | 373.2085 | 0.5 | 1.47 | 4 | 0.9 |
| VAGE | C15H26N4O7 | 375.188 | 375.1877 | 0.67 | 1.47 | 4 | 0.525 |
| GTVA | C14H26N4O6 | 347.1931 | 347.1929 | 0.54 | 1.47 | 4 | 1.225 |
| GY | C11H14N2O4 | 239.1032 | 239.1031 | 0.24 | 1.50 | 2 | -0.85 |
| AY | C12H16N2O4 | 253.1188 | 253.1187 | 0.39 | 1.50 | 2 | 0.25 |
| VVD | C14H25N3O6 | 332.1822 | 332.1821 | 0.2 | 1.54 | 3 | 1.633 |
| VV | C10H20N2O3 | 217.1552 | 217.1552 | 0.11 | 1.55 | 2 | 4.2 |
| GJQ | C13H24N4O5 | 317.1825 | 317.1825 | 0.03 | 1.55 | 3 | 0.083 |
| JVH | C17H29N5O4 | 368.2298 | 368.23 | -0.61 | 1.60 | 3 | 1.717 |
| QY | C14H19N3O5 | 310.1403 | 310.1402 | 0.43 | 1.65 | 2 | -2.4 |
| QVAA | C16H29N5O6 | 388.2196 | 388.2195 | 0.22 | 1.65 | 4 | 1.075 |
| VSGA | C13H24N4O6 | 333.1774 | 333.1773 | 0.35 | 1.66 | 4 | 1.2 |
| AGVE | C15H26N4O7 | 375.188 | 375.188 | -0.13 | 1.66 | 4 | 0.525 |
| TY | C13H18N2O5 | 283.1294 | 283.1293 | 0.33 | 1.68 | 2 | -1 |
| VPQ | C15H26N4O5 | 343.1981 | 343.1981 | 0.06 | 1.69 | 3 | -0.3 |
| TSM | C12H23N3O6S | 338.1386 | 338.1382 | 1 | 1.70 | 3 | 0.133 |
| GSY | C14H19N3O6 | 326.1352 | 326.1352 | 0.14 | 1.72 | 3 | -0.833 |
| JAD | C13H23N3O6 | 318.1665 | 318.1665 | 0.05 | 1.73 | 3 | 0.817 |
| AJ | C9H18N2O3 | 203.1396 | 203.1396 | 0.01 | 1.76 | 2 | 2.975 |
| AAVA | C14H26N4O5 | 331.1981 | 331.198 | 0.37 | 1.77 | 4 | 2.4 |
| EY | C14H18N2O6 | 311.1243 | 311.1243 | 0.15 | 1.77 | 2 | -2.4 |
| SJG | C11H21N3O5 | 276.1559 | 276.1559 | 0.12 | 1.78 | 3 | 0.983 |
| TF | C13H18N2O4 | 267.1345 | 267.1344 | 0.22 | 1.78 | 2 | 1.05 |
| VPA | C13H23N3O4 | 286.1767 | 286.1766 | 0.3 | 1.79 | 3 | 1.467 |
| FQ | C14H19N3O4 | 294.1454 | 294.1452 | 0.53 | 1.79 | 2 | -0.35 |
| VJN | C15H28N4O5 | 345.2138 | 345.2137 | 0.18 | 1.80 | 3 | 1.617 |
| SJ | C9H18N2O4 | 219.1345 | 219.1344 | 0.31 | 1.81 | 2 | 1.675 |
| TAM | C12H23N3O5S | 322.1437 | 322.1436 | 0.11 | 1.85 | 3 | 1 |
| SM | C8H16N2O4S | 237.0909 | 237.0909 | 0.04 | 1.85 | 2 | 0.55 |
| SJG | C11H21N3O5 | 276.1559 | 276.156 | -0.03 | 1.90 | 3 | 0.983 |
| TJG | C12H23N3O5 | 290.1716 | 290.1715 | 0.45 | 1.90 | 3 | 1.017 |
| GJ | C8H16N2O3 | 189.1239 | 189.1238 | 0.76 | 1.90 | 2 | 1.875 |
| NSSY | C19H27N5O9 | 470.1887 | 470.1886 | 0.17 | 1.91 | 4 | -1.6 |
| TJ | C10H20N2O4 | 233.1501 | 233.1501 | 0.21 | 1.91 | 2 | 1.725 |
| SVQE | C18H31N5O9 | 462.2210 | 462.2198 | 0.39 | 1.94 | 4 | -0.9 |
| VVA | C13H25N3O4 | 288.1923 | 288.1923 | 0.27 | 2.02 | 3 | 3.4 |
| GJ | C8H16N2O3 | 189.1239 | 189.1238 | 0.81 | 2.05 | 2 | 1.875 |
| AJE | C14H25N3O6 | 332.1822 | 332.1821 | 0.29 | 2.07 | 3 | 0.817 |
| AJT | C13H25N3O5 | 304.1872 | 304.1872 | 0.11 | 2.08 | 3 | 1.75 |
| AYA | C15H21N3O5 | 324.1559 | 324.1559 | 0.16 | 2.10 | 3 | 0.767 |
| GJE | C13H23N3O6 | 318.1665 | 318.1664 | 0.3 | 2.20 | 3 | 0.083 |
| DY | C13H16N2O6 | 297.1087 | 297.1086 | 0.23 | 2.25 | 2 | -2.4 |
| VM | C10H20N2O3S | 249.1273 | 249.1272 | 0.3 | 2.25 | 2 | 3.05 |
| TJ | C10H20N2O4 | 233.1501 | 233.1501 | 0.16 | 2.29 | 2 | 1.725 |
| PJ | C11H20N2O3 | 229.1552 | 229.1552 | 0.1 | 2.37 | 2 | 1.275 |
| JEE | C16H27N3O8 | 390.1876 | 390.1877 | -0.11 | 2.41 | 3 | -0.95 |
| AJA | C12H23N3O4 | 274.1767 | 274.1766 | 0.13 | 2.41 | 3 | 2.583 |
| VJN | C15H28N4O5 | 345.2138 | 345.2138 | 0.09 | 2.45 | 3 | 1.617 |
| JVS | C14H27N3O5 | 318.2029 | 318.2029 | 0.07 | 2.47 | 3 | 2.517 |
| ATJN | C17H31N5O7 | 418.2302 | 418.2301 | 0.14 | 2.48 | 4 | 0.438 |
| VGAE | C15H26N4O7 | 375.188 | 375.1879 | 0.32 | 2.49 | 4 | 0.525 |
| EJ | C11H20N2O5 | 261.145 | 261.1449 | 0.39 | 2.53 | 2 | 0.325 |
| AJE | C14H25N3O6 | 332.1822 | 332.1821 | 0.23 | 2.57 | 3 | 0.817 |
| SJA | C12H23N3O5 | 290.1716 | 290.1715 | 0.18 | 2.58 | 3 | 1.717 |
| SPV | C13H23N3O5 | 302.1716 | 302.1715 | 0.24 | 2.59 | 3 | 0.6 |
| VPE | C15H25N3O6 | 344.1822 | 344.1821 | 0.25 | 2.59 | 3 | -0.3 |
| FE | C14H18N2O5 | 295.1294 | 295.1294 | 0.15 | 2.61 | 2 | -0.35 |
| GJE | C13H23N3O6 | 318.1665 | 318.1665 | 0.18 | 2.63 | 3 | 0.083 |
| FS | C12H16N2O4 | 253.1188 | 253.1188 | 0.27 | 2.67 | 2 | 1 |
| SFS | C15H21N3O6 | 340.1509 | 340.1508 | 0.11 | 2.67 | 3 | 0.4 |
| NJ | C10H19N3O4 | 246.1454 | 246.1454 | 0.07 | 2.74 | 2 | 0.325 |
| YGA | C14H19N3O5 | 310.1403 | 310.1403 | 0.14 | 2.78 | 3 | 0.033 |
| APV | C13H23N3O4 | 286.1767 | 286.1766 | 0.44 | 2.79 | 3 | 1.467 |
| QJ | C11H21N3O4 | 260.161 | 260.161 | 0.1 | 2.80 | 2 | 0.325 |
| MV | C10H20N2O3S | 249.1273 | 249.1272 | 0.38 | 2.82 | 2 | 3.05 |
| SGJ | C11H21N3O5 | 276.1559 | 276.156 | -0.1 | 2.83 | 3 | 0.983 |
| VY | C14H20N2O4 | 281.1501 | 281.1501 | 0.28 | 2.83 | 2 | 1.45 |
| FA | C12H16N2O3 | 237.1239 | 237.1239 | 0.01 | 2.84 | 2 | 2.3 |
| JAT | C13H25N3O5 | 304.1872 | 304.1872 | 0.11 | 2.86 | 3 | 1.75 |
| EJE | C16H27N3O8 | 390.1876 | 390.1876 | 0.02 | 2.90 | 3 | -0.95 |
| JVS | C14H27N3O5 | 318.2029 | 318.2029 | -0.09 | 3.01 | 3 | 2.517 |
| FG | C11H14N2O3 | 223.1083 | 223.1083 | -0.26 | 3.05 | 2 | 1.2 |
| PJ | C11H20N2O3 | 229.1552 | 229.1552 | 0.19 | 3.06 | 2 | 1.275 |
| SGJ | C11H21N3O5 | 276.1559 | 276.1559 | 0.01 | 3.10 | 3 | 0.983 |
| JPT | C15H27N3O5 | 330.2029 | 330.2029 | -0.02 | 3.13 | 3 | 0.617 |
| AAY | C15H21N3O5 | 324.1559 | 324.156 | -0.02 | 3.16 | 3 | 0.767 |
| QJ | C11H21N3O4 | 260.161 | 260.161 | 0.14 | 3.16 | 2 | 0.325 |
| DPV | C14H23N3O6 | 330.1665 | 330.1665 | 0.11 | 3.18 | 3 | -0.3 |
| TVP | C14H25N3O5 | 316.1872 | 316.1872 | 0.04 | 3.20 | 3 | 0.633 |
| VVGQ | C17H31N5O6 | 402.2353 | 402.2353 | -0.01 | 3.22 | 4 | 1.125 |
| SF | C12H16N2O4 | 253.1188 | 253.1187 | 0.35 | 3.27 | 2 | 1 |
| JV | C11H22N2O3 | 231.1709 | 231.1708 | 0.4 | 3.28 | 2 | 4.175 |
| JTT | C14H27N3O6 | 334.1978 | 334.1978 | -0.04 | 3.33 | 3 | 0.917 |
| SAJ | C12H23N3O5 | 290.1716 | 290.1715 | 0.28 | 3.48 | 3 | 1.717 |
| JDA | C13H23N3O6 | 318.1665 | 318.1664 | 0.24 | 3.52 | 3 | 0.817 |
| JQA | C14H26N4O5 | 331.1981 | 331.1981 | 0.06 | 3.53 | 3 | 0.817 |
| SJEQ | C19H34N6O9 | 491.2466 | 491.2466 | -0.04 | 3.57 | 4 | -0.912 |
| DGJ | C12H21N3O6 | 304.1509 | 304.1508 | 0.06 | 3.58 | 3 | 0.083 |
| DJ | C10H18N2O5 | 247.1294 | 247.1293 | 0.34 | 3.58 | 2 | 0.325 |
| GF | C11H14N2O3 | 223.1083 | 223.1083 | 0.06 | 3.63 | 2 | 1.2 |
| VJ | C11H22N2O3 | 231.1709 | 231.1708 | 0.4 | 3.71 | 2 | 4.175 |
| MM | C10H20N2O3S2 | 281.0994 | 281.0993 | 0.2 | 3.71 | 2 | 1.9 |
| SJPG | C16H28N4O6 | 373.2087 | 373.2088 | -0.17 | 3.72 | 4 | 0.338 |
| SP | C8H14N2O4 | 203.1032 | 203.1029 | 1.17 | 3.82 | 2 | -1.2 |
| GPY | C16H21N3O5 | 336.1559 | 336.1559 | 0.18 | 3.83 | 3 | -1.1 |
| AF | C12H16N2O3 | 237.1239 | 237.1239 | 0.22 | 3.85 | 2 | 2.3 |
| GSF | C14H19N3O5 | 310.1403 | 310.1403 | 0.1 | 3.88 | 3 | 0.533 |
| QF | C14H19N3O4 | 294.1454 | 294.1454 | 0.02 | 3.93 | 2 | -0.35 |
| JSP | C14H25N3O5 | 316.1872 | 316.1872 | 0.01 | 4.02 | 3 | 0.583 |
| JPN | C15H26N4O5 | 343.1981 | 343.1982 | -0.17 | 4.04 | 3 | -0.317 |
| GJGV | C15H28N4O5 | 345.2138 | 345.2139 | -0.32 | 4.04 | 4 | 1.888 |
| VJE | C16H29N3O6 | 360.2135 | 360.2135 | -0.2 | 4.07 | 3 | 1.617 |
| VJA | C14H27N3O4 | 302.208 | 302.208 | -0.01 | 4.09 | 3 | 3.383 |
| ASJ | C12H23N3O5 | 290.1716 | 290.1716 | 0.11 | 4.10 | 3 | 1.717 |
| VGV | C12H23N3O4 | 274.1767 | 274.1766 | 0.21 | 4.11 | 3 | 2.667 |
| EGAY | C19H26N4O8 | 439.1829 | 439.183 | -0.21 | 4.14 | 4 | -0.85 |
| VJT | C15H29N3O5 | 332.2185 | 332.2186 | -0.05 | 4.14 | 3 | 2.55 |
| ADJ | C13H23N3O6 | 318.1665 | 318.1665 | 0.18 | 4.14 | 3 | 0.817 |
| FT | C13H18N2O4 | 267.1345 | 267.1344 | 0.18 | 4.16 | 2 | 1.05 |
| JV | C11H22N2O3 | 231.1709 | 231.1708 | 0.49 | 4.20 | 2 | 4.175 |
| NF | C13H17N3O4 | 280.1297 | 280.1297 | 0.1 | 4.20 | 2 | -0.35 |
| JPA | C14H25N3O4 | 300.1923 | 300.1923 | 0.16 | 4.25 | 3 | 1.45 |
| YP | C14H18N2O4 | 279.1345 | 279.1345 | 0.06 | 4.31 | 2 | -1.45 |
| AAJ | C12H23N3O4 | 274.1767 | 274.1767 | -0.19 | 4.32 | 3 | 2.583 |
| MY | C14H20N2O4S | 313.1222 | 313.1222 | 0 | 4.35 | 2 | 0.3 |
| JSP | C14H25N3O5 | 316.1872 | 316.1872 | 0.01 | 4.37 | 3 | 0.583 |
| VJA | C14H27N3O4 | 302.208 | 302.208 | -0.04 | 4.38 | 3 | 3.383 |
| AVV | C13H25N3O4 | 288.1923 | 288.1923 | 0.2 | 4.43 | 3 | 3.4 |
| SAVP | C16H28N4O6 | 373.2087 | 373.2087 | -0.06 | 4.43 | 4 | 0.9 |
| EF | C14H18N2O5 | 295.1294 | 295.1294 | -0.09 | 4.44 | 2 | -0.35 |
| SGSF | C17H24N4O7 | 397.1723 | 397.1724 | -0.12 | 4.48 | 4 | 0.2 |
| DJ | C10H18N2O5 | 247.1294 | 247.1293 | 0.46 | 4.53 | 2 | 0.325 |
| VJ | C11H22N2O3 | 231.1709 | 231.1708 | 0.45 | 4.58 | 2 | 4.175 |
| ATJ | C13H25N3O5 | 304.1872 | 304.1872 | 0.07 | 4.58 | 3 | 1.75 |
| EVM | C15H27N3O6S | 378.1699 | 378.1699 | -0.06 | 4.61 | 3 | 0.867 |
| JM | C11H22N2O3S | 263.1429 | 263.1429 | 0.1 | 4.70 | 2 | 3.025 |
| WA | C14H17N3O3 | 276.1348 | 276.1348 | 0.12 | 4.76 | 2 | 0.45 |
| YY | C18H20N2O5 | 345.145 | 345.145 | 0.18 | 4.78 | 2 | -1.3 |
| AEJ | C14H25N3O6 | 332.1822 | 332.1822 | 0.02 | 4.79 | 3 | 0.817 |
| VVVN | C19H35N5O6 | 430.2666 | 430.2668 | -0.57 | 4.88 | 4 | 2.275 |
| JPA | C14H25N3O4 | 300.1923 | 300.1923 | -0.01 | 4.89 | 3 | 1.45 |
| GTF | C15H21N3O5 | 324.1559 | 324.156 | -0.18 | 4.91 | 3 | 0.567 |
| DF | C13H16N2O5 | 281.1137 | 281.1137 | 0.15 | 4.93 | 2 | -0.35 |
| JPE | C16H27N3O6 | 358.1978 | 358.1978 | 0.05 | 4.97 | 3 | -0.317 |
| VFS | C17H25N3O5 | 352.1872 | 352.1873 | -0.11 | 5.00 | 3 | 2.067 |
| PF | C14H18N2O3 | 263.1396 | 263.1396 | 0.01 | 5.01 | 2 | 0.6 |
| JDP | C15H25N3O6 | 344.1822 | 344.182 | 0.37 | 5.03 | 3 | -0.317 |
| VVVS | C18H34N4O6 | 403.2557 | 403.2557 | -0.18 | 5.05 | 4 | 2.95 |
| SJP | C14H25N3O5 | 316.1872 | 316.1873 | -0.03 | 5.07 | 3 | 0.583 |
| PA | C8H14N2O3 | 187.1083 | 187.1081 | 0.76 | 5.08 | 2 | 0.1 |
| GYPA | C19H26N4O6 | 407.1931 | 407.1931 | -0.03 | 5.08 | 4 | -0.375 |
| JP | C11H20N2O3 | 229.1552 | 229.1552 | 0.1 | 5.11 | 2 | 1.275 |
| EEJ | C16H27N3O8 | 390.1876 | 390.1877 | -0.21 | 5.15 | 3 | -0.95 |
| JJS | C15H29N3O5 | 332.2185 | 332.2185 | 0.01 | 5.15 | 3 | 2.5 |
| EJ | C11H20N2O5 | 261.145 | 261.1451 | -0.22 | 5.17 | 2 | 0.325 |
| VJTT | C19H36N4O7 | 433.2662 | 433.2663 | -0.25 | 5.20 | 4 | 1.738 |
| APJ | C14H25N3O4 | 300.1923 | 300.1923 | 0.02 | 5.21 | 3 | 1.45 |
| JPT | C15H27N3O5 | 330.2029 | 330.2029 | 0.01 | 5.21 | 3 | 0.617 |
| ASF | C15H21N3O5 | 324.1559 | 324.156 | -0.09 | 5.22 | 3 | 1.267 |
| SVY | C17H25N3O6 | 368.1822 | 368.1822 | -0.17 | 5.28 | 3 | 0.7 |
| EAJ | C14H25N3O6 | 332.1822 | 332.1821 | 0.14 | 5.28 | 3 | 0.817 |
| VAV | C13H25N3O4 | 288.1923 | 288.1923 | 0.06 | 5.29 | 3 | 3.4 |
| JY | C15H22N2O4 | 295.1658 | 295.1657 | 0.2 | 5.31 | 2 | 1.425 |
| EFDG | C20H26N4O9 | 467.1778 | 467.178 | -0.38 | 5.33 | 4 | -1.15 |
| SW | C14H17N3O4 | 292.1297 | 292.1298 | -0.15 | 5.34 | 2 | -0.85 |
| VS | C8H16N2O4 | 205.1188 | 205.1188 | 0.19 | 5.41 | 2 | 1.7 |
| JGVS | C16H30N4O6 | 375.2244 | 375.2243 | 0.18 | 5.41 | 4 | 1.788 |
| QJE | C16H28N4O7 | 389.2036 | 389.2036 | 0.18 | 5.46 | 3 | -0.95 |
| WN | C15H18N4O4 | 319.1406 | 319.1406 | 0.21 | 5.47 | 2 | -2.2 |
| GPJ | C13H23N3O4 | 286.1767 | 286.1767 | 0.09 | 5.47 | 3 | 0.717 |
| YJA | C18H27N3O5 | 366.2029 | 366.2031 | -0.62 | 5.53 | 3 | 1.55 |
| AW | C14H17N3O3 | 276.1348 | 276.1348 | 0.12 | 5.59 | 2 | 0.45 |
| AGVY | C19H28N4O6 | 409.2087 | 409.2088 | -0.16 | 5.59 | 4 | 1.075 |
| GW | C13H15N3O3 | 262.1192 | 262.1192 | -0.18 | 5.60 | 2 | -0.65 |
| DGF | C15H19N3O6 | 338.1352 | 338.1352 | 0.02 | 5.63 | 3 | -0.367 |
| VPV | C15H27N3O4 | 314.208 | 314.208 | 0.09 | 5.66 | 3 | 2.267 |
| FV | C14H20N2O3 | 265.1552 | 265.1552 | 0.2 | 5.69 | 2 | 3.5 |
| AAF | C15H21N3O4 | 308.161 | 308.161 | 0.12 | 5.77 | 3 | 2.133 |
| TW | C15H19N3O4 | 306.1454 | 306.1454 | -0.01 | 5.78 | 2 | -0.8 |
| JJ | C12H24N2O3 | 245.1865 | 245.1864 | 0.38 | 5.80 | 2 | 4.15 |
| TPJ | C15H27N3O5 | 330.2029 | 330.2029 | -0.08 | 5.84 | 3 | 0.617 |
| TTF | C17H25N3O6 | 368.1822 | 368.1824 | -0.63 | 5.92 | 3 | 0.467 |
| SNPA | C18H31N5O6 | 414.2353 | 414.2355 | -0.57 | 5.97 | 4 | -1.025 |
| VTSJ | C18H34N4O7 | 419.2506 | 419.2508 | -0.43 | 5.97 | 4 | 1.713 |
| MJ | C11H22N2O3S | 263.1429 | 263.143 | -0.06 | 5.99 | 2 | 3.025 |
| PJN | C15H26N4O5 | 343.1981 | 343.1982 | -0.03 | 6.01 | 3 | -0.317 |
| SVJA | C17H32N4O6 | 389.24 | 389.24 | 0.09 | 6.03 | 4 | 2.338 |
| VFT | C18H27N3O5 | 366.2029 | 366.2031 | -0.54 | 6.06 | 3 | 2.1 |
| FVQ | C19H28N4O5 | 393.2138 | 393.2138 | 0 | 6.10 | 3 | 1.167 |
| MDV | C14H25N3O6S | 364.1542 | 364.1542 | 0.19 | 6.18 | 3 | 0.867 |
| TJV | C15H29N3O5 | 332.2185 | 332.2185 | 0.22 | 6.20 | 3 | 2.55 |
| GVJS | C16H30N4O6 | 375.2244 | 375.2243 | 0.07 | 6.25 | 4 | 1.788 |
| VVV | C15H29N3O4 | 316.2236 | 316.2235 | 0.34 | 6.26 | 3 | 4.2 |
| VF | C14H20N2O3 | 265.1552 | 265.1551 | 0.35 | 6.26 | 2 | 3.5 |
| VAJA | C17H32N4O5 | 373.2451 | 373.2451 | -0.13 | 6.27 | 4 | 2.988 |
| JNVE | C20H35N5O8 | 474.2564 | 474.2564 | 0.01 | 6.27 | 4 | 0.338 |
| SJV | C14H27N3O5 | 318.2029 | 318.2029 | 0.1 | 6.27 | 3 | 2.517 |
| YJ | C15H22N2O4 | 295.1658 | 295.1657 | 0.16 | 6.31 | 2 | 1.425 |
| GSJV | C16H30N4O6 | 375.2244 | 375.2243 | 0.28 | 6.44 | 4 | 1.788 |
| VFA | C17H25N3O4 | 336.1923 | 336.1923 | -0.01 | 6.45 | 3 | 2.933 |
| SJM | C14H27N3O5S | 350.175 | 350.175 | 0.04 | 6.45 | 3 | 1.75 |
| AJM | C14H27N3O4S | 334.1801 | 334.1802 | -0.39 | 6.49 | 3 | 2.617 |
| FVT | C18H27N3O5 | 366.2029 | 366.2031 | -0.43 | 6.50 | 3 | 2.1 |
| VNJ | C15H28N4O5 | 345.2138 | 345.2139 | -0.17 | 6.55 | 3 | 1.617 |
| TVJ | C15H29N3O5 | 332.2185 | 332.2184 | 0.31 | 6.59 | 3 | 2.55 |
| SVJ | C14H27N3O5 | 318.2029 | 318.2028 | 0.35 | 6.60 | 3 | 2.517 |
| VPY | C19H27N3O5 | 378.2029 | 378.2029 | 0.06 | 6.60 | 3 | 0.433 |
| JJ | C12H24N2O3 | 245.1865 | 245.1864 | 0.38 | 6.61 | 2 | 4.15 |
| GVJ | C13H25N3O4 | 288.1923 | 288.1923 | 0.27 | 6.63 | 3 | 2.65 |
| FY | C18H20N2O4 | 329.1501 | 329.1501 | 0.15 | 6.75 | 2 | 0.75 |
| AVJ | C14H27N3O4 | 302.208 | 302.2079 | 0.19 | 6.76 | 3 | 3.383 |
| GSSV | C20H30N4O7 | 439.2193 | 439.2194 | -0.34 | 6.83 | 4 | 0.55 |
| SJY | C18H27N3O6 | 382.1978 | 382.1979 | -0.22 | 6.84 | 3 | 0.683 |
| JNY | C19H28N4O6 | 409.2087 | 409.2088 | -0.3 | 6.86 | 3 | -0.217 |
| TJJS | C19H36N4O7 | 433.2662 | 433.2665 | -0.55 | 6.88 | 4 | 1.7 |
| FVGP | C21H30N4O5 | 419.2294 | 419.2294 | 0 | 6.92 | 4 | 1.25 |
| FM | C14H20N2O3S | 297.1273 | 297.1274 | -0.25 | 6.99 | 2 | 2.35 |
| JAV | C14H27N3O4 | 302.208 | 302.2079 | 0.16 | 7.10 | 3 | 3.383 |
| DVJ | C15H27N3O6 | 346.1978 | 346.1978 | -0.01 | 7.13 | 3 | 1.617 |
| VAAJ | C17H32N4O5 | 373.2451 | 373.2451 | 0.03 | 7.20 | 4 | 2.988 |
| VW | C16H21N3O3 | 304.1661 | 304.1661 | 0.04 | 7.20 | 2 | 1.65 |
| JSJ | C15H29N3O5 | 332.2185 | 332.2185 | 0.19 | 7.25 | 3 | 2.5 |
| VNJ | C15H28N4O5 | 345.2138 | 345.2139 | -0.34 | 7.29 | 3 | 1.617 |
| JJ | C12H24N2O3 | 245.1865 | 245.1864 | 0.3 | 7.29 | 2 | 4.15 |
| SJY | C18H27N3O6 | 382.1978 | 382.1978 | -0.01 | 7.29 | 3 | 0.683 |
| DVJ | C15H27N3O6 | 346.1978 | 346.1978 | 0.02 | 7.31 | 3 | 1.617 |
| APF | C17H23N3O4 | 334.1767 | 334.1766 | 0.17 | 7.38 | 3 | 1 |
| AVJ | C14H27N3O4 | 302.208 | 302.2079 | 0.16 | 7.39 | 3 | 3.383 |
| JPV | C16H29N3O4 | 328.2236 | 328.2236 | 0.23 | 7.40 | 3 | 2.25 |
| VJV | C16H31N3O4 | 330.2393 | 330.2391 | 0.51 | 7.46 | 3 | 4.183 |
| VDJ | C15H27N3O6 | 346.1978 | 346.1978 | 0.08 | 7.46 | 3 | 1.617 |
| GVVV | C17H32N4O5 | 373.2451 | 373.245 | 0.22 | 7.47 | 4 | 3.05 |
| TVPY | C23H34N4O7 | 479.2506 | 479.2505 | 0.09 | 7.48 | 4 | 0.15 |
| PY | C14H18N2O4 | 279.1345 | 279.1342 | 1.14 | 7.48 | 2 | -1.45 |
| VJS | C14H27N3O5 | 318.2029 | 318.2028 | 0.23 | 7.51 | 3 | 2.517 |
| JN | C10H19N3O4 | 246.1454 | 246.1453 | 0.43 | 7.53 | 2 | 0.325 |
| GJJN | C18H33N5O6 | 416.2509 | 416.2508 | 0.18 | 7.53 | 4 | 1.1 |
| YF | C18H20N2O4 | 329.1501 | 329.1501 | 0.24 | 7.59 | 2 | 0.75 |
| VTJ | C15H29N3O5 | 332.2185 | 332.2185 | 0.13 | 7.67 | 3 | 2.55 |
| MF | C14H20N2O3S | 297.1273 | 297.1273 | -0.05 | 7.71 | 2 | 2.35 |
| TYJ | C19H29N3O6 | 396.2135 | 396.2135 | -0.08 | 7.85 | 3 | 0.717 |
| VJV | C16H31N3O4 | 330.2393 | 330.2393 | -0.04 | 7.91 | 3 | 4.183 |
| JPY | C20H29N3O5 | 392.2185 | 392.2187 | -0.4 | 7.92 | 3 | 0.417 |
| SFVA | C20H30N4O6 | 423.2244 | 423.2245 | -0.27 | 7.96 | 4 | 2 |
| FJPS | C23H34N4O6 | 463.2557 | 463.2559 | -0.48 | 7.96 | 4 | 1.137 |
| JPV | C16H29N3O4 | 328.2236 | 328.2236 | 0.14 | 7.97 | 3 | 2.25 |
| GVF | C16H23N3O4 | 322.1767 | 322.1768 | -0.29 | 8.17 | 3 | 2.2 |
| JPY | C20H29N3O5 | 392.2185 | 392.2187 | -0.3 | 8.23 | 3 | 0.417 |
| YW | C20H21N3O4 | 368.161 | 368.161 | 0.1 | 8.24 | 2 | -1.1 |
| JF | C15H22N2O3 | 279.1709 | 279.1708 | 0.26 | 8.26 | 2 | 3.475 |
| YPJE | C25H37N5O7 | 520.2771 | 520.2772 | -0.13 | 8.28 | 4 | -0.562 |
| FGY | C20H23N3O5 | 386.1716 | 386.1716 | 0.06 | 8.30 | 3 | 0.367 |
| VVJ | C16H31N3O4 | 330.2393 | 330.2393 | 0.05 | 8.31 | 3 | 4.183 |
| FSY | C21H25N3O6 | 416.1822 | 416.1822 | -0.18 | 8.35 | 3 | 0.233 |
| SJJ | C15H29N3O5 | 332.2185 | 332.2186 | -0.02 | 8.36 | 3 | 2.5 |
| VAJ | C14H27N3O4 | 302.208 | 302.2079 | 0.16 | 8.37 | 3 | 3.383 |
| VTF | C18H27N3O5 | 366.2029 | 366.2029 | -0.05 | 8.41 | 3 | 2.1 |
| ATYJ | C22H34N4O7 | 467.2506 | 467.2507 | -0.23 | 8.48 | 4 | 0.988 |
| YAJ | C18H27N3O5 | 366.2029 | 366.2029 | -0.05 | 8.56 | 3 | 1.55 |
| VVVV | C20H38N4O5 | 415.292 | 415.292 | 0.07 | 8.62 | 4 | 4.2 |
| AJJ | C15H29N3O4 | 316.2236 | 316.2237 | -0.26 | 8.66 | 3 | 3.367 |
| SJJ | C15H29N3O5 | 332.2185 | 332.2186 | -0.02 | 8.66 | 3 | 2.5 |
| JVAV | C19H36N4O5 | 401.2764 | 401.2765 | -0.15 | 8.72 | 4 | 3.588 |
| YPJ | C20H29N3O5 | 392.2185 | 392.2186 | -0.1 | 8.73 | 3 | 0.417 |
| JW | C17H23N3O3 | 318.1818 | 318.1817 | 0.23 | 8.76 | 2 | 1.625 |
| GJJ | C14H27N3O4 | 302.208 | 302.2079 | 0.29 | 8.82 | 3 | 2.633 |
| FJ | C15H22N2O3 | 279.1709 | 279.1708 | 0.19 | 8.90 | 2 | 3.475 |
| JJV | C17H33N3O4 | 344.2549 | 344.2551 | -0.47 | 8.93 | 3 | 4.167 |
| JPJ | C17H31N3O4 | 342.2393 | 342.2393 | -0.18 | 9.11 | 3 | 2.233 |
| GYF | C20H23N3O5 | 386.1716 | 386.1717 | -0.38 | 9.13 | 3 | 0.367 |
| SJJ | C15H29N3O5 | 332.2185 | 332.2186 | -0.26 | 9.14 | 3 | 2.5 |
| AJJ | C15H29N3O4 | 316.2236 | 316.2238 | -0.58 | 9.20 | 3 | 3.367 |
| QJJ | C17H32N4O5 | 373.2451 | 373.2451 | 0.03 | 9.24 | 3 | 1.6 |
| JPJ | C17H31N3O4 | 342.2393 | 342.2394 | -0.27 | 9.28 | 3 | 2.233 |
| GJJ | C14H27N3O4 | 302.208 | 302.2079 | 0.12 | 9.30 | 3 | 2.633 |
| VJJ | C17H33N3O4 | 344.2549 | 344.2551 | -0.42 | 9.41 | 3 | 4.167 |
| VSJY | C23H36N4O7 | 481.2662 | 481.2663 | -0.18 | 9.49 | 4 | 1.562 |
| VVF | C19H29N3O4 | 364.2236 | 364.2236 | 0.13 | 9.50 | 3 | 3.733 |
| AVVJ | C19H36N4O5 | 401.2764 | 401.276 | 0.9 | 9.52 | 4 | 3.588 |
| QJJ | C17H32N4O5 | 373.2451 | 373.2451 | 0 | 9.61 | 3 | 1.6 |
| FF | C18H20N2O3 | 313.1552 | 313.1553 | -0.37 | 9.88 | 2 | 2.8 |
| VJJ | C17H33N3O4 | 344.2549 | 344.2551 | -0.56 | 9.93 | 3 | 4.167 |
| FVV | C19H29N3O4 | 364.2236 | 364.2236 | 0.05 | 10.04 | 3 | 3.733 |
| JSF | C18H27N3O5 | 366.2029 | 366.2029 | 0.06 | 10.09 | 3 | 2.05 |
| JAF | C18H27N3O4 | 350.208 | 350.2079 | 0.11 | 10.14 | 3 | 2.917 |
| WJ | C17H23N3O3 | 318.1818 | 318.1819 | -0.47 | 10.35 | 2 | 1.625 |
| GJF | C17H25N3O4 | 336.1923 | 336.1923 | 0.05 | 10.48 | 3 | 2.183 |
| JSF | C11H27N9O3S | 366.2036 | 366.2029 | 1.93 | 10.63 | 3 | 2.05 |
| GJPJ | C19H34N4O5 | 399.2607 | 399.2606 | 0.38 | 10.71 | 4 | 1.575 |
| JPF | C20H29N3O4 | 376.2236 | 376.2233 | 0.9 | 10.88 | 3 | 1.783 |
| JJJ | C18H35N3O4 | 358.2706 | 358.2706 | -0.18 | 10.92 | 3 | 4.15 |
| JJJ | C18H35N3O4 | 358.2706 | 358.2707 | -0.23 | 11.28 | 3 | 4.15 |
| DJF | C19H27N3O6 | 394.1978 | 394.198 | -0.36 | 11.29 | 3 | 1.15 |
| JJJ | C18H35N3O4 | 358.2706 | 358.2707 | -0.26 | 11.58 | 3 | 4.15 |
| FGF | C20H23N3O4 | 370.1767 | 370.1769 | -0.58 | 11.65 | 3 | 1.733 |
| AJJJ | C21H40N4O5 | 429.3077 | 429.3079 | -0.39 | 12.02 | 4 | 3.563 |
| JJF | C21H33N3O4 | 392.2549 | 392.255 | -0.11 | 12.02 | 3 | 3.7 |
| JJF | C21H33N3O4 | 392.2549 | 392.255 | -0.19 | 12.57 | 3 | 3.7 |
| JJ | C12H24N2O3 | 245.1865 | 245.1864 | 0.58 | 12.57 | 2 | 4.15 |
| JJ | C12H24N2O3 | 245.1865 | 245.1864 | 0.34 | 12.71 | 2 | 4.15 |
| AJJJ | C21H40N4O5 | 429.3077 | 429.3078 | -0.18 | 12.73 | 4 | 3.563 |

* GRAVY index score (grand average of hydrophobicity) of putatively identified short-chain peptides. A score below 0 indicates hydrophilic properties, while a score above 0 indicates hydrophobic properties. *In silico* prediction of hydrophobic properties of identified short-chain peptides was conducted using the GRAVY index (<https://www.gravy-calculator.de>).

**Table S2**. *In silico* evaluation and functional characterization of the most abundant 30 short-chain peptides annotated in infesting brown algae protein hydrolysates based on reported bioactivity, sensory attributes, antifungal potential, and free radical scavenging (FRS) capacity. Bioactivity annotations, including DPP-IV inhibitory, ACE inhibitory, antioxidant, and other reported physiological activities, were retrieved from available open-source databases, such as [BIOPEP-UWM](https://biochemia.uwm.edu.pl/biopep-uwm/?utm_source=chatgpt.com), [Bioactive Peptides Meta](http://tastepeptides-meta.com/BioactivePeptides-Meta?utm_source=chatgpt.com), and [AHTPDB](http://crdd.osdd.net/raghava/ahtpdb/pep.php?utm_source=chatgpt.com). Sensory properties (bitterness and umami) and umami intensity predictions were obtained from [TastePeptidesDB](http://tastepeptides-meta.com/TastePeptidesDB?utm_source=chatgpt.com), [Umami_YYDS](http://tastepeptides-meta.com/Umami_YYDS?utm_source=chatgpt.com), and [Umami_IP](http://tastepeptides-meta.com/Umami_IP?utm_source=chatgpt.com). Antifungal activity predictions against *C. albicans, C. krusei, C. neoformans,* and *C. parapsilosis* were generated using [AntiFungiPept](https://antifungipept.chemoinfolab.com/design/predict-activity/?utm_source=chatgpt.com), while antioxidant free radical scavenging (FRS) probabilities were predicted using [AnOxPePred 1.0](https://services.healthtech.dtu.dk/services/AnOxPePred-1.0/?utm_source=chatgpt.com).

| Peptide | DPP-IV inhibitor | ACE inhibitor | Antioxidant | Other activities | AntiOxidant  PepMeta | AHTPDB | Sensory (Biopep) | Sensory (Tastepeptides) | Bitterness/Umami | Umami Intensity Prediction | Antifungal | AFI | *C. albicans* | *C. krusei* | *C. neoformans* | *C. parapsilosis* | FRS |
| --- | --- | --- | --- | --- | --- | --- | --- | --- | --- | --- | --- | --- | --- | --- | --- | --- | --- |
| GJPJ |  |  |  |  |  |  |  |  | Bitter (1) |  | True (97.20%) | 40.32 | *99.19* | *21.95* | *17.06* | *71.16* | 0.445 |
| VJ | YES |  |  | Stimulating |  | YES | Bitter | Bitter | Bitter (1) |  | True (99.40%) | 24.99 | *7.82* | *11.81* | *53.62* | *78.78* | 0.378 |
| AJJJ |  |  |  |  |  |  |  |  | Bitter (1) |  | True (99.50%) | 19.79 | *33.18* | *23.2* | *4.91* | *40.54* | 0.34 |
| JJ | YES | YES |  | Stimulating, Lactocepin inhibitor, Inhibitor of cytosol alanyl aminopeptidase, Neuropeptide |  | YES | Bitter | Bitter | Bitter (1) |  | True (98.50%) | 17.84 | *11.66* | *22.17* | *6.29* | *62.3* | 0.39 |
| JGVS |  |  |  |  |  |  |  |  | Umami (0.9895) | 3.7432 | True (59.50%) | 51.55 | *106.89* | *30.57* | *13.59* | *159.04* | 0.348 |
| SF | YES | YES |  | Renin inhibitor | YES | YES |  | Umami | Umami (1) | 11.079 | True (86.20%) | 36.33 | *37.48* | *42.76* | *12.47* | *87.15* | 0.385 |
| ATYJ |  |  |  |  |  |  |  |  | Umami (1) | 1.3687 | True (98.60%) | 36.94 | *26.79* | *49.22* | *13.29* | *106.25* | 0.441 |
| GJJN |  |  |  |  |  |  |  |  | Bitter (0.6349) |  | True (91.70%) | 34.79 | *29.48* | *33.57* | *21.97* | *67.36* | 0.389 |
| GVJ |  |  |  |  |  |  |  |  | Umami (0.9792) | 28.8534 | True (99.10%) | 27.48 | *15.04* | *17.53* | *37.32* | *57.98* | 0.378 |
| TVPY |  |  |  |  |  | YES |  |  | Bitter (0.9997) |  | False (7.40%) | 80.89 | *84.39* | *49.01* | *60.95* | *169.79* | 0.493 |
| VSJY |  |  |  |  |  |  |  |  | Umami (0.8768) | 0.3838 | False (29.50%) | 29.37 | *38.43* | *28.2* | *4.29* | *160.16* | 0.432 |
| JNVE |  |  |  |  |  |  |  |  | Umami (1) | 0.411 | False (13.20%) | 107.26 | *72.97* | *85.51* | *107.73* | *196.88* | 0.346 |
| VV | YES | YES |  | Inhibitor of tripeptidyl peptidase II, Neprilysin inhibitor | YES | YES | Bitter, Umami | Bitter, Umami | Umami (0.5962) | 38.6236 | False (46.70%) | 40.13 | *20.72* | *24.08* | *29.78* | *174.53* | 0.36 |
| ATJN |  |  |  |  |  |  |  |  | Umami (1) | 1.3783 | False (13.20%) | 82.15 | *82.36* | *69.73* | *55.16* | *143.75* | 0.339 |
| SFS |  |  |  |  |  |  |  |  | Bitter (0.942) |  | True (86.10%) | 45.43 | *83.8* | *35.86* | *9.92* | *142.92* | 0.336 |
| SJEQ |  |  |  |  |  |  |  |  | Umami (1) | 1.1623 | False (9.70%) | 131.82 | *130.1* | *106.99* | *91.33* | *237.53* | 0.36 |
| JE |  | YES |  | Glutamate carboxypeptidase inhibitor | YES | YES | Bitter | Bitter, Umami | Bitter (1) |  | True (87.80%) | 109.74 | *56.31* | *48.02* | *177.19* | *302.68* | 0.4 |
| VY | YES | YES | YES | DPP-III inhibitor , Inhibitor of tripeptidyl peptidase 2 | YES | YES | Bitter | Bitter | Bitter (1) |  | True (99.20%) | 23.34 | *38.31* | *25.91* | *2.9* | *103.15* | 0.489 |
| SJ | YES | YES |  | regulating |  | YES | Bitter | Bitter | Bitter (1) |  | True (90.40%) | 44.67 | *34.23* | *41.4* | *21.91* | *128.21* | 0.372 |
| AVVJ |  |  |  |  |  |  |  |  | Bitter (1) |  | True (89.10%) | 38.25 | *53.47* | *22.76* | *25.38* | *69.27* | 0.312 |
| SVQE |  |  |  |  |  |  |  |  | Umami (1) | 1.17 | False (14.20%) | 116.06 | *60.22* | *73.26* | *207.6* | *198.13* | 0.352 |
| GTVA |  |  |  |  |  |  |  |  | Umami (1) | 3.9358 | True (50.90%) | 75.39 | *78.81* | *46.95* | *54.49* | *160.2* | 0.366 |
| VFA |  |  |  |  |  |  |  |  | Bitter (1) |  | True (98.30%) | 30.55 | *47.18* | *16.51* | *8.96* | *124.86* | 0.374 |
| GVVV |  |  |  |  |  |  |  |  | Bitter (1) |  | True (87.80%) | 38.45 | *37.44* | *24.25* | *22.33* | *107.77* | 0.34 |
| GJE |  |  |  |  |  |  |  | Umami | Umami (1) | 1.2764 | True (85.00%) | 123.29 | *105.41* | *70.65* | *115.39* | *268.87* | 0.4 |
| JY |  | YES | YES | Renin inhibitor | YES | YES | Bitter | Bitter | Bitter (1) |  | True (100.00%) | 17.49 | *20.02* | *23.49* | *2.65* | *74.93* | 0.502 |
| YJ | YES | YES | YES | DPP-III inhibitor, Anxiolytic peptide | YES | YES | Bitter | Bitter | Bitter (1) |  | True (99.00%) | 22.33 | *35.2* | *21.3* | *4.25* | *77.98* | 0.494 |
| AY | YES | YES | YES | Tubulin-tyrosine ligase inhibitor | YES | YES |  | Bitter | Bitter (1) |  | False (47.50%) | 29.97 | *54.71* | *39.63* | *4.2* | *88.66* | 0.492 |
| ATJ |  |  |  | Hypouricemic peptide |  |  |  |  | Umami (0.9999) | 2.7492 | True (85.30%) | 65.75 | *62.14* | *50.21* | *42.74* | *140.13* | 0.351 |
| WA | YES | YES |  | activating ubiquitin-mediated proteolysis |  | YES |  |  | Bitter (1) |  | True (92.30%) | 32.97 | *78.28* | *32.95* | *8.65* | *52.94* | 0.471 |
